# Polychlorinated Biphenyl Exposure Alters tRNA Transcriptome in High-Fat Diet-Fed Mouse Liver

**DOI:** 10.3390/ncrna11030041

**Published:** 2025-05-22

**Authors:** Carolyn M. Klinge, Julia H. Chariker, Kellianne M. Piell, Belinda J. Petri, Eric C. Rouchka, Matthew C. Cave

**Affiliations:** 1 Department of Biochemistry and Molecular Genetics, University of Louisville School of Medicine, Louisville, KY 40202, USA; kellianne.piell@louisville.edu (K.M.P.); belinda.petri@louisville.edu (B.J.P.); eric.rouchka@louisville.edu (E.C.R.); 2 Center for Integrative Environmental Health Sciences (CIEHS), University of Louisville, Louisville, KY 40292, USA; matt.cave@louisville.edu; 3 Kentucky IDeA Networks of Biomedical Research Excellence Data Science Core, University of Louisville School of Medicine, Louisville, KY 40202, USA; jhchar02@louisville.edu; 4 Department of Neuroscience Training, University of Louisville School of Medicine, Louisville, KY 40202, USA; 5 Hepatobiology and Toxicology Center, University of Louisville, Louisville, KY 40292, USA; 6 Alcohol Research Center, University of Louisville, Louisville, KY 40292, USA; 7 Superfund Research Center, University of Louisville, Louisville, KY 40292, USA; 8 Division of Gastroenterology, Hepatology & Nutrition, Department of Medicine, University of Louisville School of Medicine, Louisville, KY 40202, USA

**Keywords:** MASLD, PCBs, tRNA

## Abstract

**Background/Objectives:** Exposure of high-fat diet (HFD)-fed mice to polychlorinated biphenyls (PCBs) results in metabolic dysfunction-associated steatotic liver disease (MASLD) and progression to metabolic dysfunction-associated steatohepatitis (MASH). The mechanisms by which HFD diet and PCBs increase MASLD are unclear. Previously, we identified differences in HFD-fed mouse liver tRNA modifications with single oral exposures to the dioxin-like PCB126, the non-dioxin-like PCB mixture Aroclor 1260 (Ar1260), or the combination of Ar1260 + PCB126. **Methods:** Here, we used small RNA sequencing and the tRNA analysis of expression (tRAX) pipeline to examine if PCB exposures alter the tRNA transcriptome, including tRNA-derived fragments (tRFs), in the livers of the PCB-exposed mice. **Results:** Each PCB exposure produced distinct hepatic tRNA transcriptomes with more tRNAs decreased than increased. Only tRNA-Glu-TTC-1 was reduced with all three PCB exposures. More changes in tRFs were identified with Ar1260 alone or in combination with PCB126 than with PCB126 alone. Four tRF-3s were upregulated in both PCB126 and Ar1260 + PCB126 co-exposed mice, suggesting PCB126 as responsible for this increase. We previously reported that PCB126 exposure increased hepatic Angiogenin (ANG) protein which generates tRF-3s. Four previously reported tRNA modifications corresponded to positions of PCB-associated tRNA modifications identified by tRAX: m1A, m6A, ms2t6A, and Ψ. **Conclusions:** Overall, the differences in hepatic tRNAs and tRFs with three different PCB exposures suggest that PCB exposures play an unexplored role in regulating translation in mouse liver.

## 1. Introduction

Exposure to environmental pollutants contributes to human diseases including cancer and cardiovascular, respiratory, and neurodegenerative diseases (reviewed in [1]) and to metabolic dysfunction-associated steatotic liver disease (MASLD) [2,3,4,5]. The “exposome” that contributes to these diseases includes not only exposure to chemicals in the environment, but also lifestyle factors including diet and social/behavioral effectors. The global prevalence of MASLD, previously known as nonalcoholic fatty liver disease (NAFLD), has increased to ~25% of the population, driven largely by the rise in obesity and insulin resistance (metabolic syndrome) [6]. However, environmental exposures to pollutants acting as metabolism- and endocrine-disrupting chemicals (MDCs and EDCs), including polychlorinated biphenyls (PCBs) are associated with MASLD [5]. Self-reported workplace exposure of MASLD patients to toxicants, including arsenic, halogenated refrigerants, metals, and pesticides, was associated with a more aggressive MASLD phenotype and hepatocellular carcinoma (HCC) [4]. Recent studies have demonstrated that environmental pollutants alter chemical modifications to RNAs, referred to as the “epitranscriptome” that result in transcriptional and phenotypic changes in the livers of model organisms (reviewed in [7,8]).

tRNAs are highly abundant non-coding RNAs that are extensively modified and have rigid secondary structures allowing them to be aminoacylated (charged) with an amino acid (aa) at the 3′ end (aminoacyl-tRNA, aa-tRNA) [9]. The mouse genome contains 400 “high confidence” tRNAs decoding the standard 20 aa plus one selenocysteine tRNA: tRNA-SeC-TCA-1 [10]. The mouse genome has 47 tRNA isoacceptors: tRNA isotypes loaded with the same aa, but having a different anticodon sequence [11]. tRNA isoacceptors are expressed in a tissue-specific manner [12]. tRNAs that use synonymous codons for the same aa but different sequences in the rest of the molecule are called isodecoders [13,14].

tRNAs have the most chemical modifications of all cellular RNAs with an average of 13 modifications per eukaryotic tRNA and ~100 distinct modifications (reviewed in [9]). These modifications regulate tRNA stability, codon recognition, and the biogenesis of tRNA-derived small RNAs (tDRs). Pseudouridine (Ψ) is the most common eukaryotic tRNA modification in eukaryotes [15]. Additional common tRNA modifications include 5-methylcytosine (m5C), 7-methylguanosine (m7G), and 3-methylcytosine (m3C) additional common tRNA modifications [15]. The anticodon loop (ACL) is the site of hyper-modifications at position 34, the wobble position of the aa-charged tRNA that recognizes cognate codons in mRNA within ribosomes for translation and enhancing reading frame maintenance (reviewed in [16]). We reported that the three PCB exposures, Aroclor1260 (a mixture of non-dioxin-like (NDL) PCB congeners), PCB126 (a dioxin-like (DL) PCB congener), or the combination of Aroclor1260 + PCB126, differentially affected the liver epitranscriptome of high-fat diet (HFD)-fed male C57Bl/6J mice with changes in six post-transcriptional chemical modifications that are located in tRNAs: Am (2-O-methyladenosine), m1A (1-methyladenosine), m2,2G (N2,N2-dimethylguanosine), m5U (5-methylurdine), m6A (N6-methyladenosine), and Ψ [17].

The expression of the various specific tRNA-modifying enzymes, including methyltransferases, e.g., METTL1/2/6/8, TRMT6, and TRMT61A, are dysregulated in HCC (reviewed in [18,19]). We reported that the transcript and protein abundance of specific tRNA modifiers were altered with individual PCB exposures, e.g., TRMG61A, an m1A writer, was increased with PCB126 exposure, although m1A was reduced [20], but we did not examine the tRNA expression. The role of tRNA modifications in MASLD is currently unknown.

tDRs, also called shorter tRNA-derived small RNAs (tsRNAs) or tRNA-derived fragments (tRFs), are generated by cleavage of pre- or mature tRNAs at specific sites [21]. Hereafter, we will use the term tRFs. tRFs perform diverse biological functions and have cell- and tissue-specific expression and their expression patterns are associated with human disease (reviewed in [22,23,24,25,26,27]). Only a fraction of mature tRNAs are processed; thus, tRFs accumulate without a decline in the full-length tRNA [28]. To date, there is no uniform consensus nomenclature for tRFs. Hence, multiple names for the same or similar tRFs are found in the literature. There are six classes of tRFs: 5′-tRF, tRF-3, 3′-tRNA halves, tRF-1, internal tRFs, and “other tRFs” (reviewed in [28]). tRFs are formed by stress-induced ribonucleases including RNase I, DICER, Angiogenin (ANG), and ELAC (Elac ribonuclease Z 2) [28]. tRF-1 (tDR-1) is derived from the 3′ end of the precursor tRNA within the nucleus by ELAC cleavage [29]. tRF-5 and tRF-3 are generated by DICER and RNAse cleavage. ANG generates various tRFs, including tRF-3s, and stress-induced tRNA halves (5′-tiR and 3′-tiR) [30]. Some tRFs act like miRNAs and repress translation, although the findings are controversial [31]. A recent study identified 18 tRFs, including four alanine, two lysine, and one each of serine, arginine, histidine, and glycine tRFs, in the livers of HFD-fed (11 weeks) diet-induced obesity (DIO) C57Bl/6J mice with hepatic lipid droplet accumulation versus control mice [32]. To our knowledge, no one has examined the impact of HFD and PCB exposures on the mouse liver tRNA and/or tRF transcriptome.

In this study, we leveraged our previously generated miRNA-seq data for livers from HFD-fed C57Bl/6J male mice with vehicle control (corn oil) or a single oral exposure to Aroclor1260 or PCB126 individually or in combination [20] to identify how these PCB exposures affected the tRNA transcriptome. The livers from these PCB exposures showed lipid accumulation and fibrosis [33]. Serum markers commensurate with MASH were reported, with specific proteomic [33], miRNA, and mRNA transcriptome profiles [20], RNA modifications [17], and altered molecular pathways for each individual PCB exposure [17,20,33]. Here, we tested the hypothesis that HFD in combination with PCB exposures alters hepatic tRNA and tDR expression. We identified PCB exposure-specific unique and overlapping changes in tRNA and tDR abundance.

## 2. Results

### 2.1. PCB Exposures in HFD-Fed Mice Affect Hepatic tRNA Abundance

PCB exposures are implicated in the development of human MASLD [34]. PCBs undergo enterohepatic circulation [35]. Human exposures to potent DL PCBs, including PCB126, are only 0.02% of the total PCB exposure reported in the National Health and Nutrition Examination Survey (NHANES) database [36]. While these exposures are low, they are important due to the high potency of DL PCBs for aryl hydrocarbon receptor (AHR) activation. Because the NDL PCB mixture Ar1260 (20 mg/kg) did not activate AHR [37], PCB126 (20 µg/kg) was added to Ar1260 in our recent mouse studies [17,20,33,38,39]. The co-exposure to PCB126 + Ar1260 increases the human relevance of the administered PCB mixture. These data are the rationale for our MASLD/MASH mouse model in which male C57Bl6/J mice were fed an HFD (12 weeks) and received a single oral gavage of vehicle control (corn oil), Ar1260, PCB126, or the combination of Ar1260 + PCB126 after one week on the HFD (Figure S1). These mice had serum and hepatic changes modeling human MASH [33]. We reported large differences (99.58%) in the liver proteomes of the HFD-fed male mice exposed to Ar1260, PCB126, or the combination of Ar1260 + PCB126 [33]. In addition, liquid chromatography–mass spectrometry (LC-MS) identified seven chemical modification changes in total RNA samples from these same PCB-exposed HFD-fed mouse livers compared to HFD control [17]. These modifications are known to be in tRNAs [40].

**Figure 1:**
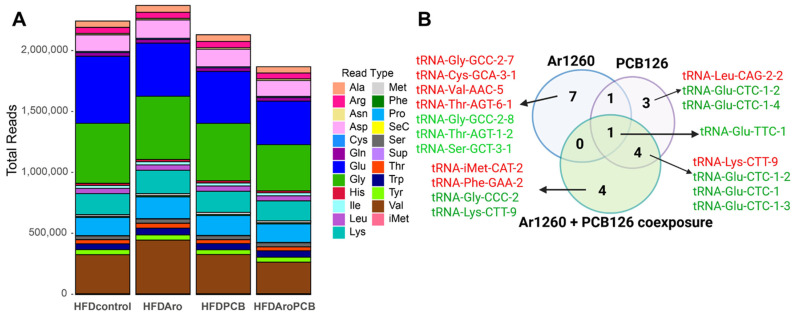
Read distribution of tRNA isotypes in the livers of the HFD-fed mice exposed to PCBs (**A**) and the impact of PCB exposures on differential tRNA abundance (**B**). All mice were fed a HFD. In A, HFDcontrol are data from the livers of the HFD-mice treated with vehicle control(corn oil); HFDAro indicates data from the HFD + Ar1260-exposed livers, HFDPCB indicates data from HFD + PCB126-exposed livers, and HFDAroPCB indicates data from the HFD + Ar1260 + PCB126 co-exposed livers samples. (**B**) The colors red and green indicate increased or decreased tRNA expression respectively in the samples as indicated. The Venn diagram was finalized using BioRender.com (accessed on 27 March 2025).

Here, we used the tRAX pipeline [41] to identify mature tRNAs and tRFs in the HFD-fed mouse livers with the three PCB exposures using our previously reported miRNA-seq data [20] (Figure S2). This analysis was performed on five separate livers (from five mice) for each of the four exposure groups: control (vehicle control), Ar1260, PCB126, and Ar1260 + PCB126. The read distribution of tRNA isotypes suggests that Ar1260 exposure increased the tRNA isotype distribution in the liver, but that PCB126 and the combination of Ar1260 + PCB126 reduced tRNA isotypes (Figure 1A).

Differential expression analysis of tRNA isodecoders was performed on the four groups of liver samples using DESeq2. There were 557 tRNAs identified, including many antisense (AS) tRNAs. Table 1, Table 2 and Table 3 show the differentially expressed isodecoder tRNAs for each PCB exposure compared to the HFD control at an FDR < 0.05. Tables S1–S3 list the antisense tRNAs (AS-tRNAs) identified for each of the three PCB exposures vs. HFD-fed mouse liver control. Table 4, Table 5 and Table 6 list the tRFs identified as differentially regulated in each of the three PCB exposures versus the HFD-fed control mouse livers. We searched the published literature for the roles of the identified tRNAs and tRFs and added this information to Table 1, Table 2, Table 3, Table 4, Table 5 and Table 6 and S1–S3.

Each of the three PCB exposures produced mostly distinct hepatic tRNA transcriptomes with little overlap (Figure 1B, Table 1, Table 2 and Table 3). Only one tRNA change was found to be in common with all three PCB exposure groups: tRNA-Glu-TTC-1 was downregulated (Figure 1B). Additional common tRNA decreases are shown. For example, tRNA-Thr-CGT-2-1 was decreased with either Ar1260 or PCB126 exposure, but not with Ar1260 + PCB126 co-exposure in vivo. Three tRNA-Glu-CTC-1 variants were decreased with both PCB126 and Ar1260 + PCB126 co-exposure. tRNA-Lys-CTT-9 was increased with PCB126 and the combined Ar1260 + PCB126 co-exposures. Table 1, Table 2 and Table 3 detail the properties of the associated amino acid (aa), indicate whether the expression of the tRNA aligns with its AS-tRNA or tRFs, and summarize additional reports pertinent to that tRNA. Four of the nine tRNAs altered with Ar1260 exposure were increased. Two of the nine tRNAs altered with PCB126 exposure were increased. Three of the eight changes in tRNAs were increased with Ar1260 + PCB126 co-exposure. Thus, the abundance of differentially expressed tRNAs was reduced overall by all three PCB exposures. There was no apparent pattern of PCB exposure, tRNA changes, and properties of the carried aa.

Glu-carrying tRNAs were reduced with all three PCB exposures. Two Thr-carrying tRNAs (tRNA-Thr-AGT-1-2 and tRNA-Thr-CGT-2-1, Table 1), tRNA-Gly-GCC-2-8, and tRNA-Ser-GCT—3-1 were decreased with Ar1260 exposure (Table 1). tRNA-Leu- CAG-2-2 and tRNA-Lys-CTT-9 were higher in PCB126-exposed livers (Table 2). With the combined Ar1260 + PCB126 exposures, tRNA-Gly-CCC-2 was decreased (Table 3) as was tRNA-Gly-GCC-2-8 with Ar1260 exposure (Table 1). tRNA-Lys-CTT-9 was increased with PCB126 exposure and in Ar1260 + PCB126 co-exposed livers (Table 2 and Table 3). tRNA-iMet-CAT-2 was increased with Ar1260 + PCB126 co-exposures. Conversely, tRNA-iMet-CAT-2_antisense was decreased with Ar1260 + PCB126 co-exposure and with Ar1260 exposure (Table 1 and Table 3).

**Figure 2:**
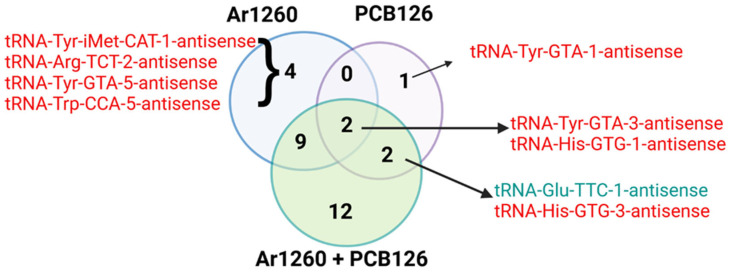
The impact of PCB exposures on tRNA-antisense abundance. The colors red and green indicate increased or decreased tRNA-antisense expression in the liver samples as indicated. The data are in the Supplementary Tables S1–S3. The Venn diagram was finalized using BioRender.com (accessed on 27 March 2025).

Figure 2 shows a Venn diagram of the tRAX-identified liver AS-tRNAs altered by the three PCB exposures vs. the HFD control (Tables S1–S3). Two AS-tRNAS, AS-tRNA-Tyr-GTA-3 and AS-tRNA-His-GTG-1, were decreased by all three PCB exposures (Figure 2). There was no association between the abundance of tRNAs and AS-tRNA in livers with Ar1260 exposure (Table 1). Of the nine tRNAs whose abundance was altered by PCB126 exposure, only one inverse association with an AS-tRNA was detected (Table 2). The abundance of tRNA-Glu-TCC-1 was reduced, but its AS-tRNA was increased by PCB126 exposure (Table 2). For the eight tRNAs altered by Ar1260 + PCB126 co-exposure, tRNA-iMET-CAT-2 and tRNA-Glu-TTC-1 showed an inverse association with their AS-tRNA, decreased and increased, respectively.

### 2.2. PCB Exposures in HFD-Fed Mice Affect Hepatic tRF Abundance

We identified 1455 tRFs in the four liver sample groups. Table 4, Table 5 and Table 6 show the differentially expressed tRFs for each PCB exposure compared to the HFD control. Eliminating the AS-tRNAs and the types of tRFs not fully annotated in tRAX [41], but leaving “other”, “partial precount”, and “wholecount” tRFs about which published tRF information [41] was available allowed us to focus our analysis (Table 4, Table 5 and Table 6). More changes in tRFs were detected with Ar1260 (Table 4) than PCB126 (Table 5). Co-exposures to Ar1260 + PCB126 resulted in the most changes in tRFs (Table 6).

**Figure 3:**
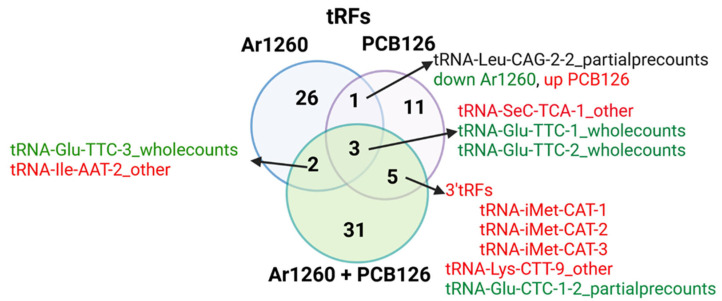
The impact of PCB exposures on differential tRF abundance in HFD-fed mouse liver. The colors red and green indicate increased or decreased tRF abundance, respectively, in the liver samples as indicated. The data are in the Supplementary Tables S1–S3. The Venn diagram was finalized using BioRender.com (accessed on 27 March 2025).

Figure 3 shows a Venn diagram of the tRAX-identified liver tRFs altered by the three PCB exposures versus the HFD control. All three PCB exposures altered three undefined tRFs. “tRNA-SeC-TCA-1_other” was increased and “tRNA-Glu-TTC-1_wholecountes”, and “tRNA-Glu-TTC-2_wholecountes” were decreased. Ar1260 and PCB126 exposures showed one common undefined tRF, “tRNA-Leu-CAG-2-2_partialprecounts”, but opposite regulation (Figure 3). Ar1260 and Ar1260 +PCB126 exposures showed two common tRFs with similar regulation: increased “tRNA-Glu-TTC-3-wholecounts” and decreased “tRNA-Ile-AAT-2_other” (Figure 3). Five tRFs, including three tRF-3s (tRNA-iMet-CAT-1, 2, and 3), showed similar regulation with PCB126 or Ar1260 + PCB126 exposures, implicating PCB126 as responsible for these changes relative to HFD alone (Figure 3). ANG is responsible for generating tRF-3s [29]. This increase in tRF-3s is in agreement with the increase in ANG protein in HFD-fed, PCB-126-exposed mouse livers [33].

**Table 4:** The differentially expressed tRFs in HFD-fed mouse liver with Ar1260 exposure vs. control (FDR < 0.05). In the column “Correspondence with tRNAs or AS-tRNAs”, “none” indicates that tRAX did not identify either a corresponding tRNA or AS-tRNA to the tRF.

tRF	Log2FC	Adj *p* Value	tRF Type	Correspondence with tRNAs (Table 1) or AS-tRNAs (Table S1)	Other Reports
tRNA-Gln-TTG-4_other	0.56	0.011	*undefined*	none	
tRNA-Leu-CAA-2-1_trailercounts	0.52	0.021	tRF-1	none	
tRNA-Val-AAC-4_fiveprime	0.51	0.011	tRF-5	none	
tRNA-Gly-TCC-1_others	0.47	0.019	*undefined*	tRNA increased; no change in AS-tRNA	tRNA-Gly-TCC-1-tRF-3 was increased in the livers of diet-induced obese (DIO) C57Bl/6 mice with lipid droplet accumulation [32].
tRNA-Pro-TGG-3_other	0.44	0.047	*undefined*	none	
tRNA-Leu-CAG-2-2_partialprecounts	0.41	0.039	*undefined*	none	
tRNA-Cys-GCA-12_other	0.40	0.040	*undefined*	none	
tRNA-Thr-AGT-6-1_trailercounts	0.39	0.037	tRF-1	none	
tRNA-Ile-AAT-2_other	0.37	0.032	*undefined*	none	
tRNA-Val-CAC-3_fiveprime	0.34	0.030	tRF-5	none	tRF-Val-CAC-005 was elevated in MASLD patient plasma and in the plasma of BALBc mice after 2–6 weeks on a high cholesterol diet [46].
tRNA-Val-AAC-2_fiveprime	0.32	0.031	tRF-5	none	
tRNA-Val-AAC-3_fiveprime	0.32	0.032	tRF-5	none	
tRNA-Val-CAC-4_fiveprime	0.30	0.024	tRF-5	none	tRF-Val-CAC-005 was elevated in MASLD patient plasma and in the plasma of BALBc mice after 2–6 weeks on a high cholesterol diet [46]
tRNA-SeC-TCA-1_other	0.30	0.042	*undefined*	none	
tRNA-Val-CAC-1_fiveprime	0.30	0.017	tRF-5	none	tRF-Val-CAC-005 was elevated in MASLD patient plasma and in the plasma of BALBc mice after 2–6 weeks on a high cholesterol diet [46].
tRNA-Val-AAC-5_fiveprime	0.28	0.042	tRF-5	tRNA-Val-AAC-5 increased; no AS-tRNA change	tRNA-Val-AAC-5-tRF-5 was increased in the livers of DIO C57Bl/6 mice [32].
tRNA-Val-AAC-5_trailercounts	0.26	0.043	tRF-1	tRNA-Val-AAC-5 increased; no AS-tRNA change	
tRNA-Gly-TCC-1_other	−0.19	0.048	*undefined*	none	tRNA-Gly-TCC-1-tRF-3 was increased in the livers of DIO C57Bl/6 mice with lipid droplet accumulation [32]
tRNA-iMet-CAT-1_other	−0.32	0.018	*undefined*	No change in tRNA; AS decreased	
tRNA-Thr-AGT-1-2_trailercounts	−0.34	0.033	tRF-1	tRNA-Thr-AGT-1-2 decreased; no AS-tRNA change	
tRNA-Leu-CAA-2_other	−0.36	0.034	*undefined*	none	tRNA-Leu-CAA was down regulated with increased ER stress [47].
tRNA-Leu-CAG-2-2_partialprecounts	−0.36	0.028	*undefined*	none	
tRNA-Leu-CAA-1_other	−0.37	0.042	*undefined*	No for tRNA, AS-tRNA was decreased	tRNA-Leu-CAA was down regulated with increased ER stress [47].
tRNA-Leu-CAG-3_other	−0.38	0.048	*undefined*	No for tRNA, AS-tRNA was decreased	
tRNA-Glu-TTC-3_wholecounts	−0.39	0.048	*undefined*	none	
tRNA-Leu-CAA-4_other	−0.39	0.031	*undefined*	none	tRNA-Leu-CAA was down regulated with increased ER stress [47].
tRNA-Leu-CAA-3_other	−0.39	0.021	*undefined*	none	tRNA-Leu-CAA is down regulated with increased ER stress [47].
tRNA-Glu-TTC-2_wholecounts	−0.41	0.025	*undefined*	none	
tRNA-Gly-GCC-2-8_trailercounts	−0.44	0.035	tRF-1	tRNA-Gly-GCC-2-7 increased; no AS-tRNA change	tRNA-Gly-GCC fragments are generated in response to activation of the ER stress response in KGN cells overexpressing IRE1α (ERN1) or in KGN, HeLa, and other cell lines treated with thapsigargin or tunicamycin [48].
tRNA-Ser-GCT-3-1_trailercounts	−0.46	0.020	tRF-1	tRNA-Ser-GCT-3-1 decreased; no AS-tRNA change	
tRNA-Glu-TTC-1_wholecounts	−0.50	0.010	*undefined*	tRNA-Glu-TTC-1 decreased; no AS-tRNA change	
tRNA-Ala-CGC-7_other	−0.62	0.001	*undefined*	none	tRF-Ala-CGC-006 was elevated in MASLD patient plasma and in the plasma of BALBc mice after 2–6 weeks on a high cholesterol diet [46]

**Table 5:** The differentially expressed tDRs in HFD-fed mouse liver with PCB126 exposure vs. control (FDR < 0.05). In the column “Correspondence with tRNAs or AS-tRNAs”, “none” indicates that tRAX did not identify either a corresponding tRNA or AS-tRNA to the tRF.

tRF	Log2FC	Adj *p* Value	Type of tRF	Correspondence with tRNAs (Table 2) or AS-tRNAs (Table S2)	Other Reports
tRNA-Leu-CAG-2-2_partialprecounts	0.59	0.003	*undefined*	tRNA-Leu-CAG-2-2 was increased; no AS-tRNA change	
tRNA-Met-CAT-1_fiveprime	0.44	0.045	tRF-5	none	
tRNA-SeC-TCA-1_other	0.374	0.012	*undefined*	none	
tRNA-SeC-TCA-1_threeprime	0.37	0.014	tRF-3	none	
tRNA-Lys-CTT-9_other	0.36	0.019	*undefined*	tRNA-Lys-CTT-9 was increased; no AS-tRNA change	
tRNA-Ser-AGA-3_fiveprime	0.35	0.040	tRF-5	none	tRNA-Ser-AGA-**1**-tRF-5 was reduced in the livers of DIO C57Bl/6 mice [32]
tRNA-iMet-CAT-1_threeprime	0.28	0.032	tRF-3	none	
tRNA-iMet-CAT-3_threeprime	0.28	0.033	tRF-3	none	
tRNA-iMet-CAT-2_threeprime	0.27	0.034	tRF-3	none	
tRNA-Leu-AAG-3_other	0.23	0.017	*undefined*	none	
tRNA-Lys-TTT-1_threeprime	0.21	0.046	tRF-3	none	tRNA-Lys-TTT-**3**-tRF-5 was increased in the livers of DIO C57Bl/6 mice [32].
tRNA-Leu-AAG-1_other	0.20	0.040	*undefined*	none	
tRNA-Leu-AAG-2_other	0.20	0.049	*undefined*	none	
tRNA-Glu-TTC-2_wholecounts	−0.36	0.049	*undefined*	none	
tRNA-Asp-GTC-1_wholecounts	−0.39	0.046	*undefined*	none	
tRNA-Glu-TTC-1_wholecounts	−0.43	0.027	*undefined*	tRNA-Glu-TTC-1 was decreased; AS-tRNA was increased	
tRNA-Glu-CTC-1-1_partialprecounts	−0.45	0.010	*undefined*	tRNA-Glu-CTC-1-1 decreased; AS-tRNA change	
tRNA-Glu-CTC-1-2_partialprecounts	−0.45	0.040	*undefined*	tRNA-Glu-CTC-1-2 decreased; AS-tRNA change	
tRNA-Glu-CTC-1-3_partialprecounts	−0.45	0.040	*undefined*	none	
tRNA-Thr-CGT-2-1_trailercounts	−0.46	0.005	tRF-1	tRNA-Thr-CGT-2-1 decreased; AS-tRNA	

**Table 6:** The differentially expressed tRFs in HFD-fed mouse liver with Ar1260 + PCB12 co-exposure vs. control (FDR < 0.05). In the column “Correspondence with tRNAs or AS-tRNAs”, “none” indicates that tRAX did not identify either a corresponding tRNA or AS-tRNA to the tRF.

tRF	Log2FC	Adj *p* Value	Type of tRF	Correspondence with tRNAs (Table 3) or AS-tRNAs (Table S3)	Other Reports
tRNA-SeC-TCA-1_other	0.66	6.76 × 10^−6^	*undefined*	none	
tRNA-iMet-CAT-3_threeprime	0.51	0.00011	tRF-3	AS decreased	
tRNA-iMet-CAT-1_threeprime	0.49	0.00018	tRF-3	none	
tRNA-iMet-CAT-2_threeprime	0.49	0.00013	tRF-3	tRNA increased; AS decreased	
tRNA-Ser-CGA-2_threeprime	0.44	0.003	tRF-3	none	
tRNA-Lys-CTT-9_other	0.44	0.004	*undefined*	tRNA increased; No change AS-tRNA	
tRNA-Glu-CTC-5_threeprime	0.40	0.029	tRF-3	none	tRNA-Glu-CTC-tRF-5 (fiveprime) was increased in exosomes purified from the blood of patients with metastatic pancreatic cancer to the liver and promoted liver metastasis by increasing the stability of WDR1 (WD Repeat Domain 1) mRNA and increasing WDR1 protein in hepatic stellate cells [49]
tRNA-Cys-GCA-5_fiveprime	0.38	0.029	tRF-5	none	
tRNA-SeC-TCA-1_threeprime	0.37	0.013	tRF-3	none	
tRNA-Ile-AAT-2_other	0.34	0.046	*undefined*	none	
tRNA-iMet-CAT-2_fiveprime	0.33	0.049	tRF-5	AS-tRNA decreased	
tRNA-Ser-GCT-4_threeprime	0.33	0.006	tRF-3	none	
tRNA-Ser-GCT-3_threeprime	0.32	0.007	tRF-3	none	
tRNA-Ser-GCT-2_threeprime	0.32	0.007	tRF-3	none	
tRNA-Arg-ACG-3_threeprime	0.30	0.0005	tRF-3	AS-tRNA decreased	
tRNA-Phe-GAA-1_other	0.30	0.0120	*undefined*	none	
tRNA-Ser-AGA-2_other	0.29	0.035	*undefined*	none	
tRNA-Arg-ACG-1_threeprime	0.28	0.001	tRF-3	AS-tRNA decreased	
tRNA-Lys-CTT-7_other	0.28	0.038	*undefined*	none	
tRNA-Ala-CGC-3_threeprime	0.28	0.038	tRF-3	none	tRF-ALA-CGC-3 was increased in DIO mouse liver [32]. tRF-Ala-CGC-006 was elevated in MASLD patient plasma and in the plasma of BALBc mice after 2–6 weeks on a high cholesterol diet [46]
tRNA-Ala-CGC-4_threeprime	0.29	0.038	tRF-3	none	tRF-Ala-CGC-006 was elevated in MASLD patient plasma and in the plasma of BALBc mice after 2–6 weeks on a high cholesterol diet [46]
tRNA-Ala-CGC-7_threeprime	0.28	0.039	tRF-3	none	tRF-Ala-CGC-006 was elevated in MASLD patient plasma and in the plasma of BALBc mice after 2–6 weeks on a high cholesterol diet [46]
tRNA-Ala-TGC-5_threeprime	0.27	0.039	tRF-3	none	
tRNA-Ala-TGC-7_threeprime	0.27	0.047	tRF-3	none	
tRNA-Ala-TGC-8_threeprime	0.27	0.046	tRF-3	none	
tRNA-Ala-TGC-6_threeprime	0.27	0.047	tRF-3	none	
tRNA-Asn-GTT-2_threeprime	0.25	0.030	tRF-3	AS-tRNA decreased	
tRNA-Phe-GAA-2_other	0.23	0.041	*undefined*	none	
tRNA-Asn-GTT-3_threeprime	0.21	0.042	tRF-3	none	
tRNA-Arg-CCG-3_threeprime	0.12	0.035	tRF-3	none	
tRNA-Lys-CTT-2_fiveprime	−0.29	0.033	tRF-5	none	
tRNA-Lys-CTT-3_fiveprime	−0.35	0.016	tRF-5	none	
tRNA-Glu-TTC-2_wholecounts	-0.37	0.045	*undefined*	none	
tRNA-Ala-CGC-7_other	−0.44	0.024	*undefined*	none	tRF-Ala-CGC-006 was elevated in MASLD patient plasma and in the plasma of BALBc mice after 2–6 weeks on a high cholesterol diet [46].
tRNA-Glu-TTC-1_wholecounts	−0.53	0.006	*undefined*	none	
tRNA-Gly-CCC-2_fiveprime	−0.54	0.013	tRF-5	AS-tRNA increased	tRF-5 tRNA-Gly-CCC-2 was upregulated and positively correlated with the inflammation level and ANG expression in mouse tibialis anterior (TA) muscle after injury from injection of 50 μL of 10 μΜ cardiotoxin [50].
tRNA-Glu-CTC-1-1_partialprecounts	−0.57	0.008	*undefined*	none	
tRNA-Glu-CTC-1-2_partialprecounts	−0.57	0.008	*undefined*	none	
tRNA-Glu-CTC-1-3_partialprecounts	−0.57	0.008	*undefined*	none	
tRNA-Glu-TTC-3_wholecounts	−0.63	0.001	*undefined*	none	
tRNA-Glu-CTC-1_wholecounts	−0.64	0.0006	*undefined*	none	

**Figure 4:**
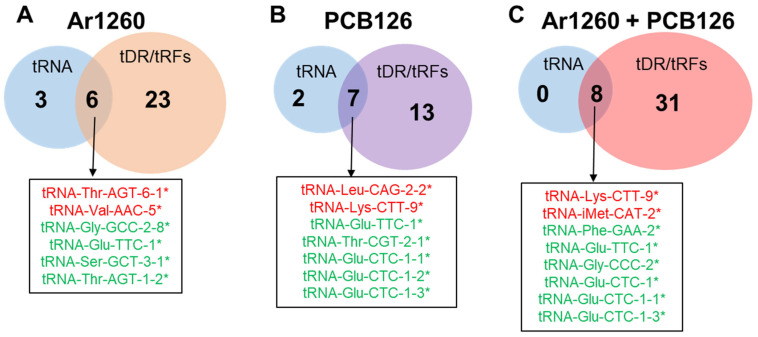
Association of changes in the abundance of tRNAs and tRFs in HFD-fed mouse liver with PCB exposures. These are tRNAs and tDRs that were differentially expressed in HFD-control versus the indicated PCB exposures. (**A**) Ar1260 exposure; (**B**) PCB126 exposure; (**C**) Ar1260 + PCB126 coexposures. The colors red and green indicate increased or decreased tRNA expression in the samples as indicated. * indicates the same directional change(increased or decreased) of the corresponding tRF(Table 4, Table 5 and Table 6).

Since tRNAs and their tRFs are expected to correlate in terms of direction of expression [51], we examined the data in Venn diagrams (Figure 4). In the Ar1260-exposed livers, two tRNAs that were increased relative to the HFD control showed a corresponding increase in tRFs: tRNA-Thr-AGT-6-1 and tRNA-Val-AAC-5 (Table 1 and Table 4). Similarly, four downregulated tRNAs also showed decreased tRF abundance (Table 1 and Table 4). These concordant results suggest a correlation between these tRNAs and their tRFs in the livers after Ar1260 exposure. With PCB126 exposure, two tRNAs, tRNA-Leu-CAG-2-2 and tRNA-Lys-CTT-9, showed increased abundance and a corresponding increase in tRFs (Table 2 and Table 5). Five tRNAs were decreased with PCB126 exposure and corresponding tRFs were also decreased (Table 2 and Table 5). However, tRNA-Glu-TTC-1 abundance was decreased with PCB126 exposure, whereas a corresponding tRF and the AS-tRNA were increased (Table 2). For the co-exposure to Ar1260 + PCB126, all eight tRNA changes showed concordant changes in tRF abundance (Table 3, Figure 4). Both upregulated tRNAs and all six tRNAs downregulated with Ar1260 + PCB126 co-exposure also showed increased and reduced tRF abundance, respectively (Table 3, Figure 4). Again, the corresponding increases or decreases in tRNAs and their tRFs suggests concordant tRNA processing.

However, not all of the detected changes in tRFs were associated with changes in the abundance of parental tRNAs. For example, tRAX detected an increase in tRNA-SeC-TCA-1 fragments with Ar1260, PCB126, and the Ar1260 + PCB126 exposures. However, no change in intact tRNA-Sec-TCA-1 was detected in these samples. Likewise, we observed an increase in tRNA-Met-CAT-1-tRF-5 and tRNA-Met-CAT-1,2,3-tRF-3 with PCB126 and Ar1260 + PCB126 co-exposures, but no increase in mature tRNA-Met-CAT-1 was detected.

### 2.3. PCB Exposures in HFD-Fed Mice Affect Hepatic tRNA Modifications Detected by RT Misincorporation Terminations by tRAX

tRAX generated a plot of the per-base mismatch frequencies in each tRNA isodecoder, as a dot plot comparison across samples (Figure S5). tRNA positions 6, 9, 24, 25, 26, 32, 34, 37, 56, 58, and 59 showed the widest misincorporations among the tRNA isotypes, implying chemical modifications at those base positions. We previously identified seven PCB exposure-associated modification changes known to be located in tRNAs [52] in total RNA samples isolated from HFD-fed mouse livers [17]. We compared that list of PCB-induced tRNA modifications with the tRNA positions known to contain those modifications and the potential impact on tRF formation. One caveat is that because of the current lack of high throughput-verified detection, “only a small fraction of tRNA modifications have been studied: m5C, m1A, and Ψ” [28]. Four tRNA modifications that correspond to positions of PCB-associated misincorporations were identified: m1A, m6A, ms2t6A, and Ψ. m1A has been identified at positions 9, 14, 22, 57, and 58 in tRNAs [53]. N6-threonylcarbamoyladenosine (t6A) is the most common modification of position 37 in tRNA [40]; however, we did not detect any differences in t6A in Ar1260-, PCB126-, or Ar1260 + PCB126-exposed samples versus control mouse liver samples [17].

## 3. Discussion

The prevalence of MASLD is increasing [54]. Chronic nutrient overload is a starting point for insulin resistance with impaired glucose and lipid metabolism in the liver, resulting in steatosis initiating the spectrum of MASLD [55]. In addition to obesity, other contributors to MASLD include genetic variants (SNPs) [56], epigenetics [57,58], gut microbiome dysbiosis [59,60,61], and exposure to metabolism-disrupting environmental chemicals (MDCs), e.g., PCBs [34].

Here, we used the tRAX pipeline [41] to identify changes in the tRNA and tRF transcriptome in the livers of HFD-fed male mice exposed to Ar1260, an NDL PCB mixture, PCB126, a DL PCB, and the combination of Ar1260 + PCB126 [33]. These mimic PCB exposures in human populations that are associated with liver abnormalities and metabolic disease [36,62,63]. Our studies have demonstrated mostly unique results with few overlapping changes in proteins [33], mRNA [20], miRNA [20], and RNA modifications [17] in response to these three PCB exposures. We identified for the first time common and unique changes in tRNA and tRF abundance with each of the three PCB exposures. The biological significance of these PCB-exposure-induced changes in the hepatic tRNA transcriptome lies in their potential to disrupt translation processes, ultimately leading to alterations in protein expression.

### 3.1. tRNAs

Glu-carrying tRNAs were reduced with Ar1260, PCB126, and Ar1260 + PCB126 exposures. Glu is a regulator of hepatic aa metabolism that inhibits inflammatory responses in the liver and has antioxidant activity [64]. We reported that these PCB exposures contribute to MASH development in HFD-fed male mice [33]. Exposures to PCB126, Ar1260, or both Ar1260 + PCB126 did not exacerbate the HFD-induced liver lipid accumulation in the mice [33]. While we did measure Glu (or any other aa) in the liver samples, decreased hepatic Glu increases fat accumulation [64]. Two leu-carrying tRNAs were increased in PCB126-exposed livers. PCB126-exposed mice had lower plasma ALT levels compared to Ar1260-exposed mice and co-exposure Ar1260 + PCB126 attenuated ALT levels compared to Ar1260 alone, suggesting that PCB126 may protect against liver injury [33]. Leu is an essential branched-chain aa (BCAA) metabolized in the liver that decreases hepatic steatosis [64]. Two val-carrying tRNAs were increased with Ar1260 exposure. Val is another essential BCAA that is decreased in liver cirrhosis [65]. We examined the protein abundance of hepatic Glu-metabolizing EPRS1 protein (gene Eprs1, bifunctional glutamate/proline--tRNA ligase), but no change was detected with the PCB exposures [33]. We did not detect protein changes in BCAA-metabolizing enzymes BCKD (branched-chain alpha-ketoacid dehydrogenase kinase), BCAT1 (branched-chain-amino-acid aminotransferase, cytosolic), or BCAT2 (branched-chain-amino-acid aminotransferase, mitochondrial) in the livers of the HFD-fed mice with any of these three PCB exposures [33].

PCB126 and Ar1260 + PCB126-exposed mouse livers showed a decrease in tRNA-Glu-TTC-1, suggesting a PCB126-mediated reduction. Knockdown of human XPOT (exportin for tRNA) increased nuclear tRNA-Glu-TTC-1 in human triple-negative breast cancer (TNBC) cell lines [42]. Exportin hepatic protein abundance was decreased with Ar1260 + PCB126 co-exposure, but no change was detected with PCB126 exposure [33]. Theoretically, decreased exportin protein would be expected to increase nuclear retention of tRNA-Glu-TTC-1. Because small RNAs were extracted from the mouse livers in this study using the miRNAeasy kit which includes both nuclear and cytosolic small RNAs [20], no association appears related to a decrease in both exportin and tRNA-Glu-TTC-1 with Ar1260 + PCB126 co-exposure. Future studies could examine the subcellular distribution of tRNA-glu-TTC-1 in PCB126 or Ar1260 + PCB126-exposed HFD-fed mouse livers.

Ar1260 + PCB126 co-exposure increased hepatic tRNA-iMet-CAT-2. tRNA-iMet initiates protein synthesis, a finding corresponding to the higher number of hepatic proteins detected with Ar1260 + PCB126 co-exposure compared to either Ar1260 or PCB126 exposure alone [33]. Concordantly, the gene ontology (GO) process “regulation of protein metabolic processes” was increased by Ar1260 + PCB126 co-exposure [33], suggesting the need for increased tRNA-iMet-CAT for initiation of translation.

Ar1260 exposure decreased two Thr-carrying tRNAs. One of these tRNAs, tRNA-Thr-CGT-2-1, was also decreased with PCB126 exposure. No Thr-carrying tRNAs were altered in the Ar1260 + PCB126 co-exposure livers. Thr is an essential aa catabolized in the liver [64]. Neither of the hepatic Thr-metabolizing enzymes (TDG, G/T mismatch-specific thymine DNA glycosylase or TDH, L-Threonine dehydrogenase) were altered by any of the three PCB exposures in our HFD-fed mouse model of MASLD/MASH [64].

Ar1260 + PCB126 co-exposure decreased one hepatic gly-carrying tRNA and a different gly-carrying tRNA was decreased with Ar1260 exposure. Gly is a non-essential aa that regulates bile synthesis [66]. Gly was increased in mouse livers on an HFD [67]. Oral gly prevents liver fibrosis in rats [68]. We did not detect any change in the protein levels of SHMT1 (serine hydroxymethyltransferase, cytosolic, which converts serine and tetrahydrofolate to gly and 5,10-methylene tetrahydrofolate) in the livers of any of the three groups of PCB-exposed mice [33]. Likewise, no change in AGT1 (alanine-glyoxylate aminotransferase, Agxt (gene) which converts glycoxylate to gly) protein was detected with these PCB exposures [33].

PCB126 and Ar1260 + PCB126 exposures increased one hepatic lys-carrying tRNA, suggesting regulation by PCB126. Lys is an essential aa that is catabolized in the liver [69]. We did not detect any protein change in LKR/SDH (L-lysine-2-ketoglutarate reductase, Aass (gene), which converts lys to saccharopine, or ALDH7A1 (alpha-aminoadipic semialdehyde dehydrogenase), which converts saccharopine to glutamate [69], in the livers of the HFD-fed mice with these PCB exposures [33].

Taken together, these data suggest that PCB-exposure-induced changes in liver aa-carrying tRNAs are not reflected in alterations in the protein abundance of enzymes involved in their respective hepatic aa metabolism. We did not detect any protein changes in the 29 aa transporters expressed in the liver [70] in response to any of the three PCB exposures in the HFD-fed mouse livers [33]. Uncharged tRNAs accumulate in mammalian cells when aa are limited, trigging the integrated stress response (ISR) [71]. GO process analysis of the proteins altered by these PCB exposures did not indicate IRS [33]. A future goal is to perform metabolic flux analysis using ^13^C- labeled aa and mouse livers ex vivo [72] to examine how HFD and the three PCB exposures in vivo impact aa metabolism.

### 3.2. AS-tRNAs

For the majority of tRNAs, there was no correspondence with change in its AS-tRNA. We note that the role of AS-tRNA in mouse liver is unclear. We only found one paper in PubMed [73] reporting AS-tRNAs. In that study, the authors identified sense and antisense mt-tRNAs with “mirrored” expression of some mite *T. urticae* mt-tRNAs, i.e., tRNA-Arg, tRNA-Glu, tRNA-Leu1, tRNA-Phe, tRNA-Pro, tRNA-Ser2, and tRNA-Tyr, other tRNAs had high-antisense and low-sense tRNA expression, whereas some tRNAs had high-sense and low-antisense reads [73]. We suggest that the application of targeted small RNA sequencing using Illumina technology enabled the detection of AS-tRNA. Further studies are needed on the role of AS-tRNAs in mammals and in MASLD.

### 3.3. tRFs

The rapid advancement in RNA-seq technologies has identified novel tRNA transcriptome species including tRFs that have roles in cellular biology [74]. Here, we found that PCB exposures increased the abundance of more tRFs than tRNAs. For tRNAs altered by PCB exposures with a corresponding tRF, the tRNA and tRF showed parallel regulation—both increased or decreased. This result supports the expected concordant regulation of the “parental” tRNA and its cleavage to tRFs. However, not all tRNAs showed changes in tRFs. For example, a tRF for tRNA-Leu-CAA-2 was decreased with Ar1260 exposure. However, no change in parental tRNA-Leu-CCA-2 was detected. The opposite patterns of tRNA and tRF and AS-tRNA abundance may relate to PCB-induced alterations in RNA-binding proteins, elongation factors, or ribosomal proteins that may protect some tRNA species from degradation [75,76,77]. tRNA-Leu-CAA is downregulated with endoplasmic reticulum (ER) stress [47]. However, proteome analysis did identify changes in protein abundance in the ER stress pathway [33].

Some of the changes in hepatic tRFs detected here in response to PCB exposures (Table 4, Table 5 and Table 6) were also reported in the steatotic livers of DIO-induced C57BL/6J mice [32], in human MASLD patient plasma [46], and in mouse plasma after 2–6 weeks of high-cholesterol diet-induced MASLD in BALBc mice [46]. For example, tRFs from tRNA-Gly-TCC-1 and tRNA-Val-CAC were increased with Ar1260 exposure. This observation agrees with the increase in tRNA-Gly-TCC-1-tRF-3 and tRNA-Val-CAC in the livers of DIO C57Bl/6 mice [32] and the plasma of MASLD patients [46], respectively. We detected increased tRF-5 fragments from tRNA-Val-AAC with Ar1260 exposure, but not with PCB126 exposure, which also apparently blocked tRNA-Val-AAC cleavage when combined with Ar1260 exposure. tRNA-Val-AAC-5-tRF-5 was increased in the livers of DIO C57Bl/6J mice [32]. Increased tRNA-Val-AAC fragments were detected in mouse liver samples with time after liver isolation and in human HepG2 cells subjected to stressors including starvation, heat shock, hypothermia, hypoxia, and radiation [78]. 5′-tRFs from tRNA-Val inhibit eiF4F complex formation [28]. Interestingly, this corresponds to our observation that Ar1260 exposure produced the fewest changes in the hepatic proteome [33].

However, not all tRF changes agree with these reports. The tRF “tRNA-Gly-TCC-1_other” was lower with Ar1260 exposure but tRNA-Gly-TCC-1-tRF-3 was increased in the steatotic livers of DIO C57Bl/6 mice [32]. This difference may result from the co-exposures to PCBs with HFD to induce MASLD and progression to MASH in our mouse model.

The tRNA endonuclease ANG is highly expressed in liver and generates tRF-3 and 5′tiRFs [79]. We reported that ANG protein was increased in HFD-fed, PCB126-exposed mouse livers [33]. However, we did not detect altered mouse liver protein abundance of DICER or ELAC2 with any of the three PCB exposures [33]. DICER and ELAC2 are responsible for generating tRF-5s and tRF-1 [80,81]. Likewise, no change in IRE1α (ERN1, serine/threonine-protein kinase/endoribonuclease IRE1α) protein that generates tRNA-Gly-GCG-5′tRFs in response to ER stress in cells and in mouse ovary [48] was detected in the HFD-fed, PCB-126 exposed mouse livers [33].

tRFs regulate translation, interact with DNA in regulating transcription, and are mediators in RNA interference, like miRNAs [28]. Here, we observed that PCB126 exposure increased tRFs from tRNA-Lys. tRFs from tRNA-Lys associate with Argonaute 2 (AGO2) [28]. We observed the fewest hepatic miRNA changes with PCB126 exposure in HFD-fed mouse liver [20]. Future studies will be necessary to examine the identities of liver AGO2-associated tRFs and miRNAs and the impact of HFD and PCB exposures on these interactions.

tRNA modifications generally suppress tRF biogenesis [28]. m5C and Ψ were associated with tRF biogenesis [28]. ALKBH3-mediated demethylation of m1A in tRNA makes tRNAs more sensitive to ANG cleavage and tRF generation [80]. Since ANG protein was increased in the PCB126-exposed mouse livers [33], it is possible that the decrease in m1A in PCB126-exposed liver samples [17] is associated with the increase in the five tRF-3s in those samples. However, overall, it is difficult to draw conclusions associating positions of modifications in tRNAs with tRFs identified in our PCB-exposed liver samples. Ongoing efforts in direct-read RNA technologies and bioinformatic analysis will be needed to understand how tRNA modifications affect the tRNA transcriptome.

## 4. Conclusions

Using tRAX [41], we identified changes in tRNAs and tRFs (tDRs) with PCB exposures in HFD-fed male mouse livers. Our study provides the first evidence of differences in the abundance of individual tRNAs in the livers of HFD-fed male C57Bl/6J mice with three exposures (Ar1260, PCB126, or Ar1260 + PCB126). These findings suggest that PCB exposures play an unexplored role in regulating translation. Further, we report for the first time HFD- and PCB-exposure-induced changes in the abundance of tRFs which may regulate transcript abundance and translation as well as additional processes including extracellular vesicles for communication with other tissues [82]. We speculate that HFD and PCB exposures act as hepatic stressors to generate differences in tRFs. Changes in the hepatic pool of tRNAs is likely to cause differences in translation and thus protein abundance. Additionally, changes in tRNAs can lead to errors in tRNA loading in ribosomes, which decreases the accuracy and efficiency of protein synthesis, leading to mutated proteins with altered function [83]. Future studies will examine if tRNAs associate with mistranslated (altered) proteins in this HFD-fed PCB-exposure model of MASLD progression to MASH. It will be of interest to examine if tRFs are altered in the blood and extracellular vesicles of humans with PCB and other environmental exposures associated with metabolic diseases including MASLD.

### Limitations

tRNA structure and modifications affect cDNA synthesis in the RT step prior to RNA-seq, thus reducing the efficiency and accuracy of tRNA-seq analysis [84]. We did not treat the isolated miRNA samples with ALKB or T4 polynucleotide kinase that increase cDNA reads [41]. The presence of tRFs can interfere with estimating the mature tRNA pool [51]. Lastly, we did not explore the mechanism of functional implications of our results.

## 5. Materials and Methods

### 5.1. Animal Studies

The experimental design is modeled in Figure S1. The mouse protocol for PCB exposure and HFD was ratified by the University of Louisville Institutional Animal Care and Use Committee (Approval code 18022 on 30 March 2018) [33] and details of this study have been previously reported [33]. In brief, male C57BL/6 mice (8 weeks old) from Jackson Laboratory were randomized (*n* = 10) into four equal groups. All mice were fed ad libitum a high-fat diet (HFD, 15.2, 42.7, and 42.0% of total calories from protein, carbohydrate, and fat, respectively; TekLad TD88137) throughout the study. At ten weeks of age, each mouse was given a one-time, single oral gavage of either corn oil (vehicle control), Ar1260 (20 mg/kg), PCB126 (20 μg/kg), or a mixture of Ar1260 (20 mg/kg) plus PCB126 (20 μg/kg). These concentrations were selected based on our previous reports that Ar1260 and PCB126 act as “second hits” in HFD-fed mice to induce steatohepatitis [38]. Since humans are exposed to dioxin-like (DL) and non-dioxin-like (NDL) PCB mixtures, PCB126 (a DL-PCB) was added to Ar1260 at doses used in our previous studies [37,38]. At the end of week 12, the mice were fasted for ~ 6 h prior to euthanasia and liver samples were harvested as described [33].

### 5.2. Short RNA Sequencing

Short RNAs were isolated from five mouse livers/experimental exposure using Qiagen miRNA kits and sequenced as previously described [85]. Briefly, libraries were prepared from 1 µg of mouse liver RNA. The QIAseq miRNA Library Kit (Qiagen) and quality and quantity were validated using an Agilent Bioanalyzer (Agilent Technologies, Santa Clara, CA, USA). The RNA Integrity Number (RIN) for the samples is shown in Table S4. Figure S6 shows a linear regression analysis of RIN vs. read count from the Illumina miRNA-seq data, which shows that higher RIN corresponds with lower read count. Lower RIN scores were not associated with loss of miRNA detection [86]. Sequencing library quantitation was carried out by performing a MiSeq Nano (Illumina, San Diego, CA, USA) test run. Two sequencing runs/sample were performed on the Illumina NextSeq 500 using the NextSeq 500/550 High Output Kit v2.5 (75 cycles). Data as fastq files were downloaded from Illumina’s BaseSpace onto the KY INBRE server for analysis. The raw data of the miRNA-seq are available at the Gene Expression Omnibus (GEO) database: GSE195829.

### 5.3. Bioinformatics Analysis

Twenty fastq files (four exposures groups: control, Ar1260, PCB126, Ar1260 + PCB126, with five mouse livers per exposure group) were trimmed to remove the Qiagen 3′ Adapter sequence. Quality control (QC) of the raw sequence data was performed using FastQC (version 0.10.1). Minimum quality values for all samples were well above Q30 (1 in 1000 error rate); therefore, no quality trimming was necessary. The number of sequenced reads ranged from 4.4 to 6.9 million across the samples with a median of 5.8 million. Raw fastq files were input to tRAX (tRNA analysis of expression), a tool for identifying tRNAs and tDRs/tRFs and performing differential expression between comparison groups [35,40]. Supplementary Figure S2 shows the tRAX pipeline in which trimmed fastq files are aligned with the Bowtie2 aligner [36,40] prior to abundance estimation and differential expression. For details regarding tRAX’s alignment options, which are specifically designed to take into account the characteristics of tRNAs, see [40]. Sequenced reads were aligned to the *Mus musculus* (mm10) genome assembly. The alignment rate was high, ranging from 91.7 to 96.2 percent across the samples with a median of 95.7 percent. The pipeline relied on the creation of a mouse tRNA database that included a tRNA scan file generated by tRNAscan-SE [37,40] and downloaded from GtRNAdb [11,38,40] as well as a name map file that converted the tRNAscan-SE IDs to GtRNAdb gene symbols. The tRNA identification and differential expression analysis were performed using a tRAX Python (version 2.7.5) script called processsamples.py. The script was run with the --no frag option to identify mature tRNAs and without the option to identify tRFs. tRAX uses DESeq2 for differential expression analysis which utilizes a negative binomial distribution appropriate for count data. Raw counts are input to DESeq2 and normalized with the relative log expression (RLE) method prior to performing differential expression. A false discovery rate (FDR) of 5% was used to determine significance and reduce the number of false positives in the results. tRAX performed a principal component analysis (PCA, see Figure S7) as part of its pipeline. The samples show wide within-group variability.

tRAX provided a report containing the percentage of reads retained after adapter removal, read mapping rate, proportion of reads mapped to tRNAs versus rRNAs and unannotated regions, percentage of expected read lengths, and range of read count normalization size factors across samples. In the analysis of mature tRNAs, all quality filters were passed with the exception of tRNA read share greater than 50% and at least 70% of reads between 40 and 75 bases in length. In the analysis of tRFs, all filters were passed with the exception of at least 70% of reads between 15 and 50 bases. The read counts, mapping rate, and percentages for these latter filters are shown in Table S5. Reads that aligned to mouse tRNA gene loci with flanking sequences are considered as pre-tRNA reads and were not included in the analysis of mature tRNAs. Supplementary Figure S3 displays the distribution of reads for tRNAs and other small RNA types across samples. Reads referred to as “other” are not mapped to any annotated features.

### 5.4. Venn Diagrams

The Venn diagrams were drawn using http://bioinformatics.psb.ugent.be/webtools/Venn/. (accessed on 3 April 2025)

## Figures and Tables

**Table 1:** Differentially expressed mature, nuclear-encoded tRNAs in HFD-fed mouse liver with Ar1260 exposure vs. control (FDR < 0.05). In the column “Correspondence with tRFs”, “undefined” indicates that tRAX did not define the type of tRF.

tRNA	Log2FC	Adj *p* Value	Properties of Carried aa	Correspondence with AS-tRNA (Table S1)	Correspondence with tRFs (Table 4)	Other Reports
tRNA-Gly-GCC-2-7	0.51	0.016	Non-polar, hydrophobic	no	no	
tRNA-Thr-AGT-6-1	0.41	0.039	Polar, uncharged, hydrophilic	no	Yes: tRF-1 Increased	
tRNA-Cys-GCA-3-1	0.29	0.035	Polar, hydrophobic	no	no	
tRNA-Val-AAC-5	0.27	0.024	Non-polar, hydrophobic	no	Yes: tRF-5 and undefined; Both were increased.	tRNA-Val-AAC-5-tRF-5 was increased in the livers of DIO C57Bl/6J mice [32].
tRNA-Glu-TTC-1	−0.28	0.039	Polar, acidic, hydrophilic	no	Yes: undefined Decreased	Increased nuclear tRNA-Glu-TTC-1 in triple-negative breast cancer cell lines MDA-MB-231 and MDA-MB-268 after stable human XPOT (exportin for tRNA) expression [42].
tRNA-Thr-AGT-1-2	−0.31	0.046	Polar, hydrophilic, uncharged	no	Yes, tRF-1 Decreased	
tRNA-Thr-CGT-2-1	−0.34	0.043	Polar, hydrophilic, uncharged	no	Yes, tRF-1 Decreased	
tRNA-Gly-GCC-2-8	−0.44	0.041	Non-polar hydrophobic	no	Yes, tRF-1 Decreased	
tRNA-Ser-GCT-3-1	−0.46	0.027	Polar, hydrophilic	no	Yes: undefined Decreased	

**Table 2:** Differentially expressed mature, nuclear-encoded tRNAs in HFD-fed mouse liver with PCB126 exposure vs. control (FDR < 0.05). In the column “Correspondence with tRFs”, “undefined” indicates that tRAX did not define the type of tRF.

tRNA	Log2FC	Adj *p* Value	Properties of aa	Correspondence with AS-tRNA (Table S2)	Correspondence with tRFs (Table 5)	Other Reports
tRNA-Leu-CAG-2-2	0.62	0.003	Non-polar, hydrophobic	no	Yes: undefined, Increased	
tRNA-Lys-CTT-9	0.38	0.021	Polar, charged, basic	no	Yes: undefined, Increased	
tRNA-Glu-CTC-1	−0.31	0.049	Polar, charged, acidic	no		
tRNA-Glu-TTC-1	−0.32	0.018	Polar, charged, acidic	Yes: opposite direction: AS- tRNA-Glu-TTC-1 abundance was increased	Yes, undefined, Increased	Increased nuclear tRNA-Glu-TTC-1 was reported in triple-negative breast cancer cell lines MDA-MB-231 and MDA-MB-268 after stable knockdown of human XPOT (exportin for tRNA) [42].
tRNA-Glu-CTC-1-4	−0.35	0.048	Polar, acidic, hydrophilic	no	no	
tRNA-Thr-CGT-2-1	−0.45	0.006	Polar, hydrophilic, uncharged	no	Yes, tRF-1 Decreased	Upregulated in HEK-293 cells when RPB1 is down regulated and cells are treated with DRB to block RNA Pol II pause-release, thus blocking RNA pol II activity [43].
tRNA-Glu-CTC-1-3	−0.55	0.017	Polar, charged, acidic	no	Yes, undefined Decreased	
tRNA-Glu-CTC-1-1	−0.55	0.017	Polar, charged, acidic	no	Yes, undefined Decreased	
tRNA-Glu-CTC-1-2	−0.55	0.017	Polar, charged, acidic	no	Yes, undefined Decreased	

**Table 3:** Differentially expressed mature, nuclear-encoded tRNAsin mouse liver with HFD + Ar1260 + PCB126 combination exposure vs. HFD control (FDR < 0.05). In the column “Correspondence with tRFs”, “undefined” indicates that tRAX did not define the type of tRF.

tRNA	Log2FC	Adj *p* Value	Properties of aa	Correspondence with AS-tRNA (Table S3)	Correspondence with tRFs (Table 6)	Comments and Published Reports
tRNA-Lys-CTT-9	0.46	0.004	Polar, charged, basic	no	Yes, undefined Increased	
tRNA-iMet-CAT-2	0.31	0.016	Non-polar, hydrophobic	Yes, but opposite direction: decreased tRNA-iMet-CAT-2_antisense abundance	Yes, tRF-3 Increased	Methylated G at position 9 [44]. Not regulated by SOX4 [45].
tRNA-Phe-GAA-2	0.26	0.036	Non-polar, aromatic side chain, hydrophobic	no	Yes, undefined Increased	
tRNA-Glu-TTC-1	−0.39	0.004	Polar, charged, acidic	Yes but opposite direction: increased tRNA-Glu-TTC-1_antisense	Yes, undefined Decreased	Increased nuclear tRNA-Glu-TTC-1 was reported in triple-negative breast cancer cell lines MDA-MB-231 and MDA-MB-268 after stable knockdown of human XPOT (exportin for tRNA) [42].
tRNA-Gly-CCC-2	−0.47	0.017	Non-polar, hydrophobic	no	tRF-5 Decreased	
tRNA-Glu-CTC-1	−0.57	0.0002	Polar, charged, acidic	no	Yes, undefined Decreased	
tRNA-Glu-CTC-1-3	−0.63	0.006	Polar, charged, acidic	no	Yes, undefined Decreased	
tRNA-Glu-CTC-1-1	−0.63	0.006	Polar, charged, acidic	no	Yes, undefined Decreased	

## Data Availability

The raw data of the miRNA-seq is available at Gene Expression Omnibus (GEO) database: GSE195829.

## Supplementary Materials

The following supporting information can be downloaded at: https://www.mdpi.com/article/10.3390/ncrna11030041/s1, Figure S1: Short-term (12 weeks) PCB-exposure in a HFD-fed mouse model of MASH. Figure S2: The tRAX pipeline used to analyze the miRNA-seq liver samples from HFD-fed control, Ar1260, PCB126, and Ar1260 + PCB126 exposed mice. Figure S3. Percentage of uniquely mapped (single), multimapped (mult) and unmapped (unmap) reads for each sample (A) and percentage of uniquely mapped tRNAs, unique_anticodons (equal matches to multiple different tRNAs with the same anticodon), and unique_aminos (equal matches to tRNAs decoding the same amino acid) (B). Figure S4: PCB exposures differentially regulate mouse liver antisense-tRNA. Figure S5. Overall maximum per-base mismatch frequency across all samples. Figure S6: Linear regression analysis of mouse liver miRNA RIN and Read Count from Illumina miRNA-seq analysis. Figure S7: Principal component analysis (PCA) of the tRNA transcriptome for the mouse liver samples. Each point represents the data from one individual mouse liver sample. All mice were fed a HFD and exposed to vehicle control (HFDcontrol), Ar1260 (HFDAro), PCB126 (HFDPCB), or Ar1260 + PCB126 co-exposures (HFDAroPCB). Table S1: Differentially expressed antisense (AS)-tRNAs in HFD-fed mouse liver with Ar1260 exposure vs. HFD-fed control (FDR < 0.05). Table S2: Differentially expressed antisense (AS)-tRNAs in HFD-fed mouse liver with PCB126 exposure vs. HFD-fed control (FDR < 0.05). Table S3: Differentially expressed antisense (AS)-tRNAs in HFD-fed mouse liver with Ar1260 + PCB126 exposure vs. HFD-fed control (FDR < 0.05). Table S4: The RNA Integrity Number (RIN) for the isolated miRNA samples from mouse livers in the four treatment groups indicated. Table S5: Read counts, mapping rate, and tRAX quality measure not meeting expected criteria for some or all samples. References [20,32,46,47,87,88] are cited in the supplementary materials.

## Abbreviations

The following abbreviations are used in this manuscript:

PCB	polychlorinated biphenyls
HFD	high-fat diet
MASLD	metabolic dysfunction-associated steatotic liver disease
MASH	metabolic dysfunction-associated steatohepatitis
tDRs	tRNA-derived small RNAs
tRF	tRNA-derived fragment
tsRNAs	shorter tRNA-derived small RNAs
AS-tRNA	antisense tRNA
NDL	non-dioxin like
DL	dioxin-like
AHR	arylhydrocarbon receptor
ANG	angiogenin
MDC	metabolism-disrupting chemicals
EDC	endocrine-disrupting chemicals
HCC	hepatocellular carcinoma
NAFLD	nonalcoholic fatty liver disease
ACL	anticodon loop
Am	2-O-methyladenosine
m1A	1-methyladenosine
m2,2G	N2,N2-dimethylguanosine)
m3C	3-methylcytosine
m5C	5-methylcytosine
m5U	5-methylurdine
m6A	N6-methyladenosin
Ψ	pseudouridine
ELAC	Elac ribonuclease Z 2
DIO	diet-induced obesity
ER	endoplasmic reticulum
UPR	unfolded protein response
