# Supplementary material for: Polychlorinated Biphenyl Exposure Alters tRNA Transcriptome in High-Fat Diet-Fed Mouse Liver
Source: Noncoding RNA. 2025 May 22;11(3):41. doi: 10.3390/ncrna11030041 (PMC12195632; doi:10.3390/ncrna11030041)
Supplement: Supplementary file 1 [file ncrna-11-00041-s001.zip › ncrna-3597975-supplementary.pdf]

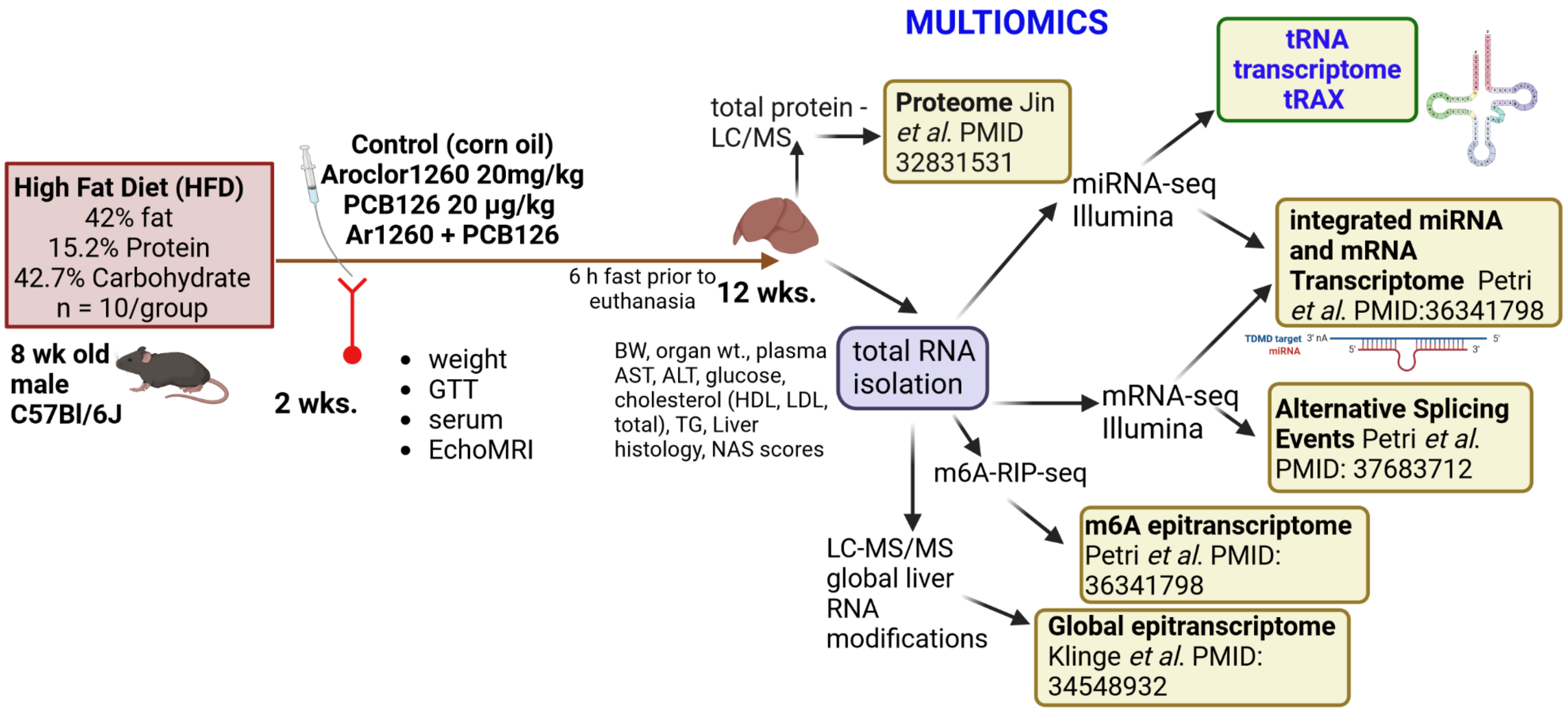

Figure S1: Short-term (12 wks.) PCB-exposure in a HFD-fed mouse model of MASH. Male C57Bl/6J mice were randomized into four groups (n=10/group). All mice were fed a HFD (TekLad TD88137) prior to and during this study. After 2 wks., the mice were given corn oil (control), Aroclor1260 (20 mg/kg), PCB126 (20 µg/kg), or Aroclor1260 + PCB126 via a one-time gavage and followed for 10 additional wks. on the HFD. Livers were harvested after euthanasia. Proteins were extracted from the livers and LC/MS measured peptide abundance. RNA was isolated with Trizol, rRNA depleted, and polyA-selected for mRNA seq using TruSeq Stranded mRNA Library Prep Kit with short read RNA-seq on the Illumina NextSeq 500. m6A-RIP-seq was performed to identify m6A peaks in gene transcripts on the Illumina NextSeq 500. Global epitranscriptomics was performed by LC-MS/MS. miRNA was isolated using Qiagen miRNA kits and miR-seq performed using QIAseq miRNA Library Kit with miRNA-seq performed on Illumina the NextSeq 500. The miRNA-seq data in GEO GSE195829 was the starting point of the tRNA transcriptome analysis reported here. The Figure was created using Biorender.com.

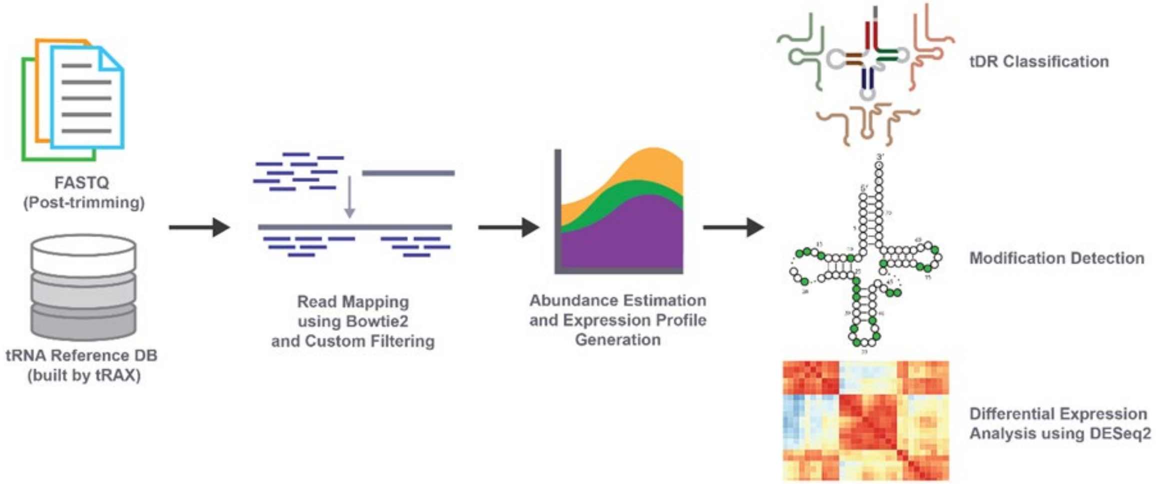

Figure S2: The tRAX pipeline used to analyze the miRNA-seq liver samples from HFD-fed control, Ar1260, PCB126, and Ar1260+PCB126 exposed mice to identify the tRNA transcriptome.

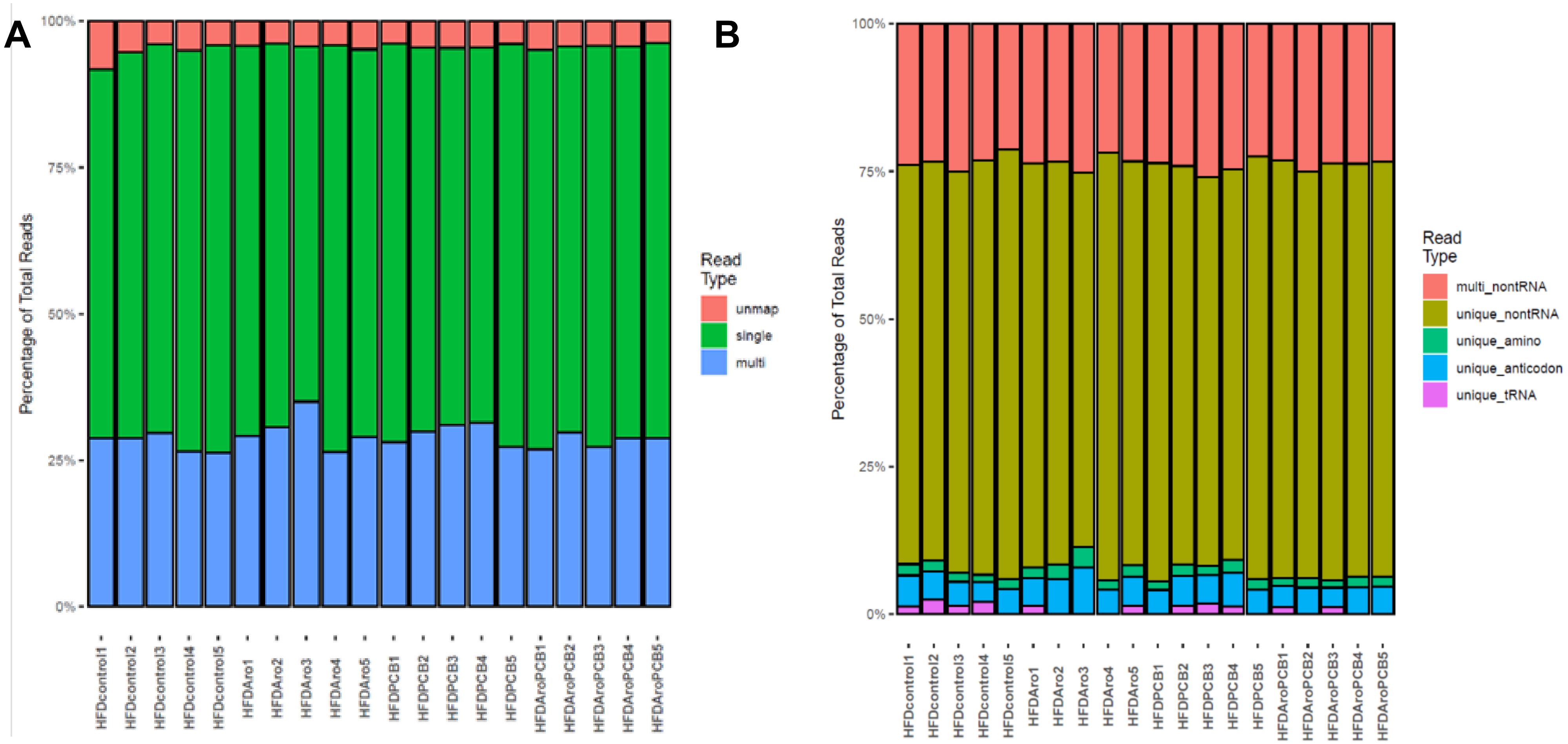

Figure S3. Percentage of uniquely mapped (single), multimapped (mult) and unmapped (unmap) reads for each sample (A) and percentage of uniquely mapped tRNAs, unique_anticodons (equal matches to multiple different tRNAs with the same anticodon), and unique_aminos (equal matches to tRNAs decoding the same amino acid) (B). This analysis was performed in tRAX. All mice were fed a HFD and exposed to vehicle control (HFDcontrol), Ar1260 (HFDAro), PCB126 (HFDPCB), or Ar1260 + PCB126 co-exposures (HFDAroPCB).

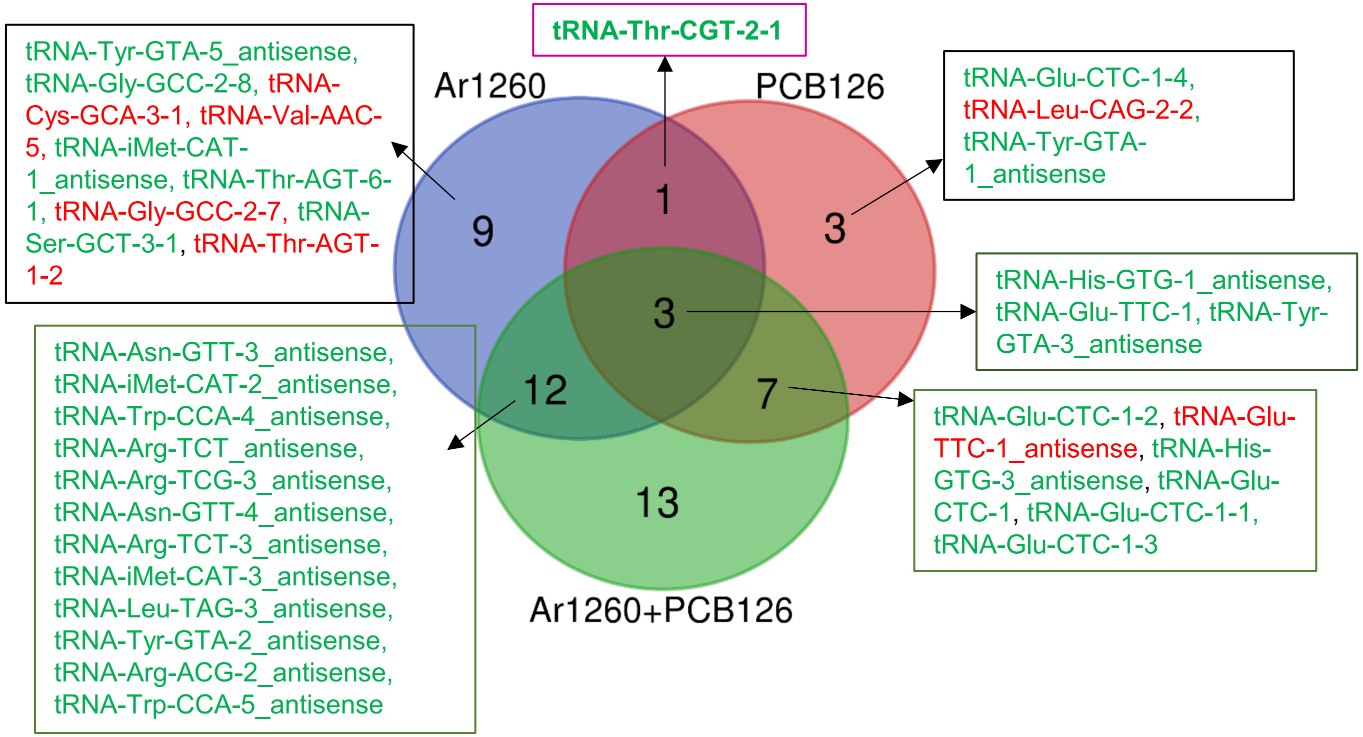

Figure S4. PCB exposures differentially regulate mouse liver antisense-tRNA expression. Shown are the number of AS-tRNAs differentially expressed between the HFD-fed control mouse livers and the HFD-fed PCB-exposed mouse livers (Ar1260, PCB126, or Ar1260 + PCB126 co-exposure). AS-tRNAs were identified by tRAX. The colors red and green indicate increased or decreased tRNA expression in the samples as indicated.

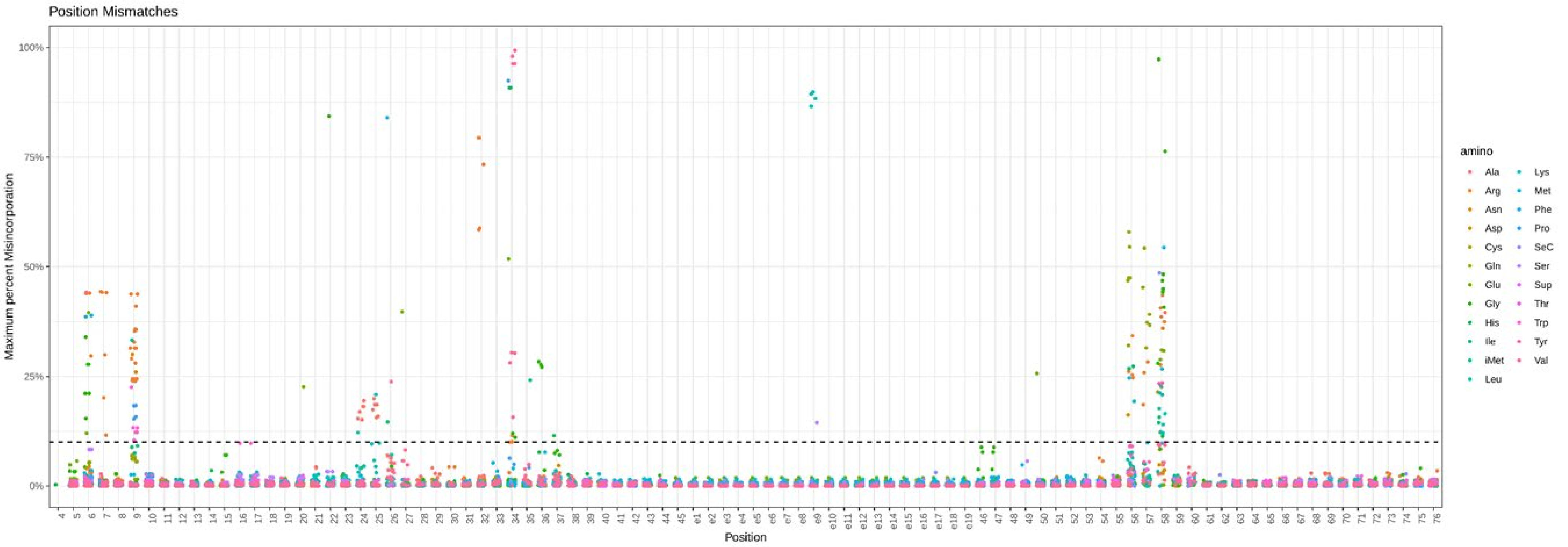

Figure S5: Overall maximum per-base mismatch frequency across all samples. tRAX generated a plot of the Per-base mismatch frequencies in each tRNA isodecoder, a dot plot comparison across samples.

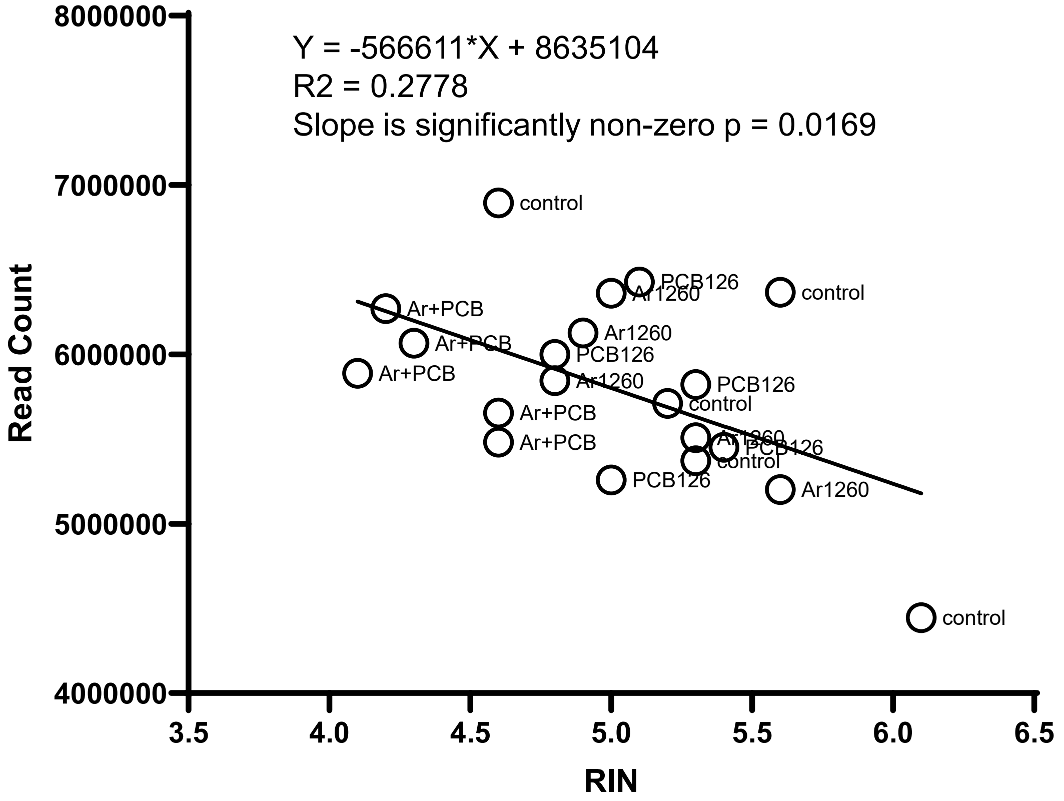

Figure S6: Linear regression analysis of RIN and Read Counts form mouse liver miRNA-seq from Illumina miRNA-seq analysis. The data were analyzed in GraphPad Prism and the equation, r2, and, significance of the slope are included within the graph. Each point is labeled for the individual mouse treatments. Each point represents the data from one individual mouse liver sample.

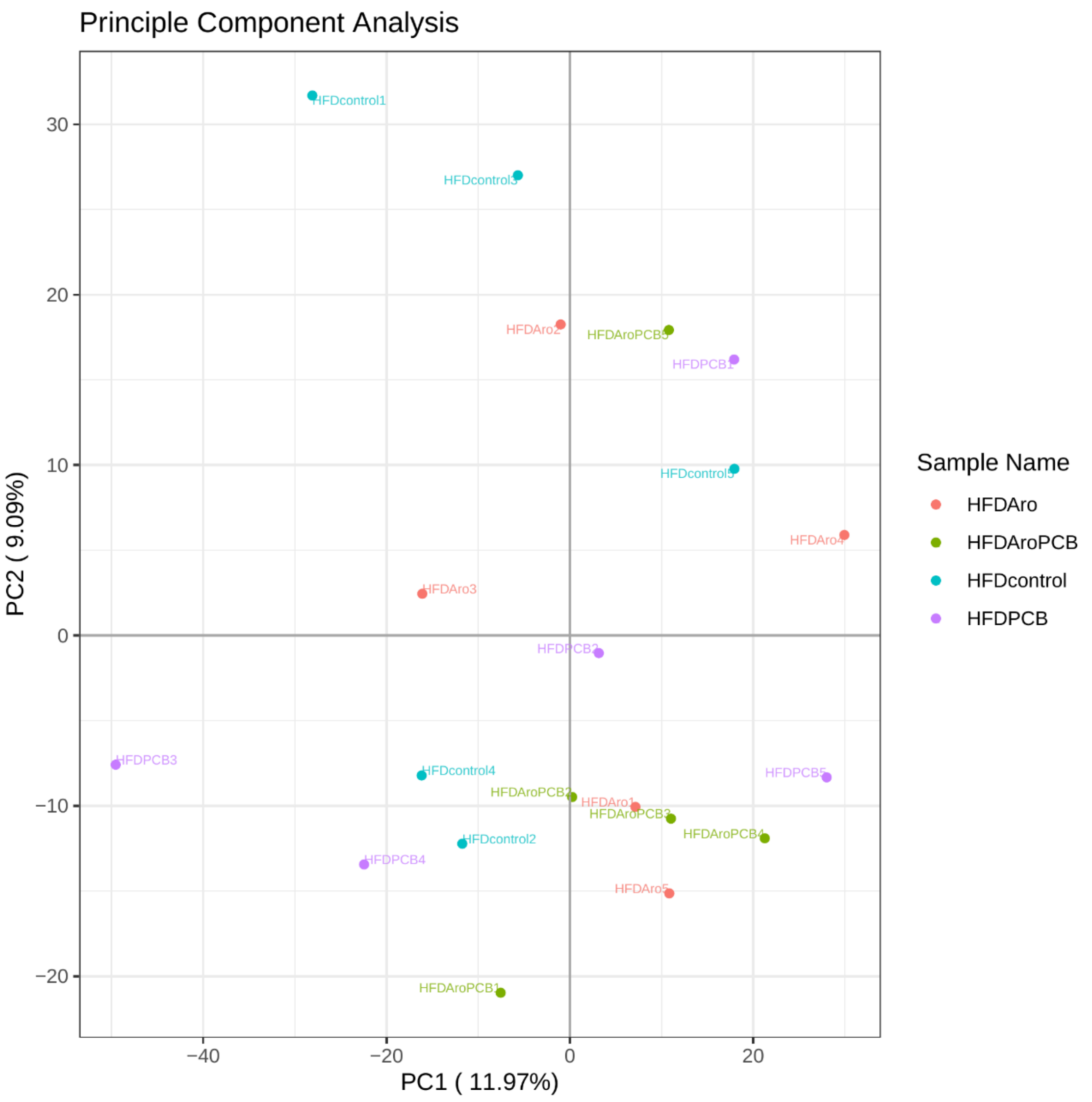

Figure S7: Principal component analysis (PCA) of the tRNA transcriptome for the mouse liver samples. Each point represents the data from one individual mouse liver sample. All mice were fed a HFD and exposed to vehicle control (HFDcontrol), Ar1260 (HFDAro), PCB126 (HFDPCB), or Ar1260 + PCB126 co-exposures (HFDAroPCB).

Table S1: Differentially expressed antisense (AS)-tRNAs in HFD-fed mouse liver with Ar1260 exposure vs HFD-fed control (FDR < 0.05). In the column Correspondence with tRFs, "undefined" indicates that tRAX did not define the type of tRF and the relative abundance of the tRF is indicated compared to the Log2FC of the AS-tRNA

AS-tRNA	Log2FC	Adj p value	Correspondence with tRFs (Table 4)	Other reports
tRNA-Trp-CCA-5_antisense	-0.38	0.048	none	
tRNA-iMet-CAT-2_antisense	-0.39	0.043	none	
tRNA-Arg-TCT-3_antisense	-0.46	0.027	none	
tRNA-Tyr-GTA-5_antisense	-0.466	0.045	none	tRNA-Tyr-GTA was down regulated with increased ER stress (1).
tRNA-Asn-GTT-4_antisense	-0.466	0.026	none	
tRNA-Trp-CCA-4_antisense	-0.47	0.011	none	
tRNA-Arg-ACG-2_antisense	-0.47	0.025	none	
tRNA-His-GTG-1_antisense	-0.47	0.036	none	tRF-His-GTG-001 was elevated in MASLD patient plasma His-GTG (2). tRNA-His-GTG-tRF-1 was increased in the livers of DIO C57Bl/6J mice (3).
tRNA-iMet-CAT-1_antisense	-0.51	0.009	Yes, undefined; Decreased	
tRNA-Tyr-GTA-3_antisense	-0.54	0.021	none	tRNA-Tyr-GTA is down regulated with increased ER stress (1). HFD (12 wk) induced the ER stress response protein pathway in C57Bl/6J male mice (4).
tRNA-Arg-TCT-2_antisense	-0.55	0.007	none	
tRNA-Leu-TAG-3_antisense	-0.57	0.006	none	
tRNA-iMet-CAT-3_antisense	-0.61	0.002	none	
tRNA-Arg-TCG-3_antisense	-0.62	0.005	none	
tRNA-Asn-GTT-3_antisense	-0.63	0.002	none	

Table S2: Differentially expressed antisense (AS)-tRNAs in HFD-fed mouse liver with PCB126 exposure vs HFD-fed control (FDR < 0.05). In the column Correspondence with tRFs, “undefined” indicates that tRAX did not define the type of tRF and the relative abundance of the tRF is indicated as relative compared to the Log2FC of the AS-tRNA (decreased or increased).

AS-tRNA	Log2FC	Adj p value	Correspondence with tRFs (Table 5)	Other reports
tRNA-Glu-TTC-1_antisense	0.42	0.042	Yes, undefined: Decreased	
tRNA-His-GTG-1_antisense	-0.47	0.034	none	tRF-His-GTG-001 was elevated in MASLD patient plasma His-GTG (2). tRNA-His-GTG-tRF-1 was increased in the livers of DIO C57Bl/6J mice (3).
tRNA-Tyr-GTA-1_antisense	-0.49	0.034	none	tRNA-Tyr-GTA is down regulated with increased ER stress (1).
tRNA-His-GTG-3_antisense	-0.50	0.030	none	tRF-His-GTG-001 was elevated in MASLD patient plasma (2)
tRNA-Tyr-GTA-3_antisense	-0.55	0.019	none	tRNA-Tyr-GTA is down regulated with increased ER stress (1).

Table S3: Differentially expressed antisense (AS)-tRNAs in HFD-fed mouse liver with Ar1260 + PCB126 exposure vs HFD-fed control (FDR < 0.05). In the column Correspondence with tRFs, "undefined" indicates that tRAX did not define the type of tRF and its relative abundance is indicated as relative compared to the Log2FC of the AS-tRNA (decreased or increased).

AS-tRNA	Log2FC	Adj p value	Correspondence with tRFs (Table 6)	Other reports
tRNA-Glu-TTC-1_antisense	0.45	0.030	none	
tRNA-Asn-GTT-1_antisense	-0.40	0.028	Yes, undefined: Decreased	
tRNA-Trp-CCA-4_antisense	-0.42	0.023	none	
tRNA-Ala-AGC-5_antisense	-0.42	0.045	none	
tRNA-Thr-AGT-5_antisense	-0.42	0.039	none	
tRNA-Arg-ACG-3_antisense	-0.44	0.045	Yes, tRF3: Increased	
tRNA-Trp-CCA-5_antisense	-0.45	0.021	none	
tRNA-Arg-TCT-3_antisense	-0.46	0.026	none	
tRNA-Arg-ACG-2_antisense	-0.46	0.027	none	
tRNA-His-GTG-3_antisense	-0.47	0.042	none	
tRNA-Arg-TCG-3_antisense	-0.47	0.032	none	
tRNA-Asn-GTT-4_antisense	-0.48	0.022	none	
tRNA-Tyr-GTA-3_antisense	-0.48	0.038	none	
tRNA-Leu-CAG-3_antisense	-0.49	0.033	none	
tRNA-His-GTG-1_antisense	-0.50	0.024	none	
tRNA-Leu-CAA-1_antisense	-0.51	0.026	none	
tRNA-Asn-GTT-2_antisense	-0.52	0.014	Yes, tRF-3: Increased	
tRNA-Arg-TCT-4_antisense	-0.52	0.017	none	
tRNA-iMet-CAT-2_antisense	-0.55	0.005	Yes, tRF-5: Increased	
tRNA-Trp-CCA-2_antisense	-0.58	0.005	none	
tRNA-Tyr-GTA-2_antisense	-0.58	0.013	none	tRNA-Tyr-GTA is down regulated with increased ER stress (1).
tRNA-iMet-CAT-3_antisense	-0.58	0.003	Yes, tRF-5: Increased	
tRNA-Trp-CCA-1_antisense	-0.61	0.0023	none	
tRNA-His-GTG-1_antisense	-0.63	0.00233	none	tRF-His-GTG-001 was elevated in MASLD patient plasma and in the plasma of BALBc mice after 2-6 wks on a high cholesterol diet (2). tRNA-His-GTG-tRF-1 was increased in the livers of DIO C57Bl/6J mice (3).
tRNA-Asn-GTT-3_antisense	-0.66	0.001244	Yes, tRF-3: Increased	

tRNA-Leu-TAG-3_antisense	-0.85	4.57E-05	none	

Table S4: The RNA Integrity Number (RIN) for the isolated miRNA samples from mouse livers in the four treatment groups indicated. Each sample is from one mouse. The RIN values in the Ar1260 + PCB126 co-exposure samples was significantly lower than the HFD control, Ar1260, and PCB126 samples (one-way ANOVA followed by Tukey's multiple comparison test with p values < 0.01, 0.05, and 0.05, respectively (data from GraphPad Prism analysis not shown). miRNA-seq was run on an Illumina NextSeq 500 using the NextSeq 500/550 High Output Kit v2.5 (75 Cycles) (5). Data as fastq files were analyzed for quality control (QC) by FastQC (6) as previously reported (5). All samples were considered of sufficient quality and did not require quality trimming sine the values for all 20 samples was well above Q30 (1 in 1000 error rate).

**Mouse liver sample (all mice were fed a HFD)**	**Sample #**	**RIN from ABI Bioanalyzer**
HFD Control	1	6.1
HFD Control	2	4.6
HFD Control	3	5.2
HFD Control	4	5.6
HFD Control	5	5.3
Ar1260	1	4.9
Ar1260	2	5.6
Ar1260	3	5.3
Ar1260	4	5.0
Ar1260	5	4.8
PCB126	1	5.4
PCB126	2	5.3
PCB126	3	5.1
PCB126	4	5.0
PCB126	5	4.8
Ar1260 + PCB126	1	4.2
Ar1260 + PCB126	2	4.6
Ar1260 + PCB126	3	4.3
Ar1260 + PCB126	4	4.1
Ar1260 + PCB126	5	4.6

Table S5. Read counts, mapping rate, and tRAX quality measures. Starred items did not pass filters. These RNA-seq data are from liver samples from five individual male C57Bl/6J mice fed a HFD for 12 wks. with a single oral exposure to vehicle control (corn oil) (HFDcontrol), Aroclor1260 (20 mg/kg) (HFDAro), PCB126 (20 µg/kg) (HFBPCB), or the combination of the doses of Aroclor1260 (20 mg/kg) + PCB126 (20 µg/kg) (HFDAroPCB). The raw data of the miRNA-seq is available at Gene Expression Omnibus (GEO) database: GSE195829.

Sample	Read Count	Mapping Rate	tRNA Read Percentage	Mature tRNA: Percentage reads between 40 and 75 bases	tDR: Percentage reads between 15 and 50 bases
HFDcontrol1	4,444,576	91.76%	*8.64%	*0.61%	74.72%
HFDcontrol2	6,894,325	94.78%	*9.32%	*0.55%	*66.25%
HFDcontrol3	5,711,426	96.00%	*7.16%	*0.62%	71.28%
HFDcontrol4	6,367,728	94.97%	*6.85%	*0.68%	*66.31%
HFDcontrol5	5,372,772	95.93%	*7.18%	*0.46%	*56.08%
HFDAro1	6,127,233	95.77%	*8.16%	*0.60%	*65.62%
HFDAro2	5,202,404	96.18%	*9.31%	*0.41%	*68.26%
HFDAro3	5,508,924	95.66%	*12.19%	*0.44%	81.26%
HFDAro4	6,360,875	95.94%	*6.78%	*0.53%	*57.8%
HFDAro5	5,844,731	95.19%	*8.63%	*0.58%	*61.45%
HFDPCB1	5,451,489	96.18%	*6.61%	*0.53%	*60.78%
HFDPCB2	5,821,484	95.49%	*8.63%	*0.57%	*68.06%
HFDPCB3	6,427,133	95.45%	*8.27%	*0.87%	75.22%
HFDPCB4	5,258,249	95.48%	*9.47%	*0.62%	71.60%
HFDPCB5	6,000,455	96.09%	*7.17%	*0.49%	*54.87%
HFDAroPCB1	6,270,080	95.14%	*6.25%	*0.83%	*65.1%
HFDAroPCB2	5,481,580	95.68%	*7.07%	*0.63%	*69.04%
HFDAroPCB3	6,066,036	95.81%	*5.92%	*0.62%	*59.35%
HFDAroPCB4	5,888,538	95.61%	*7.56%	*0.57%	*54.64%
**HFDAroPCB5**	5,654,254	96.26%	*7.25%	*0.43%	*66.57%

References Cited:

1. Guillon J, Coquelet H, Leman G, Toutain B, Petit C, Henry C, Boissard A, Guette C, Coqueret O. tRNA biogenesis and specific aminoacyl-tRNA synthetases regulate senescence stability under the control of mTOR. PLOS Genetics 2021; 17:e1009953
2. Huang P, Tu B, Liao H-j, Huang F-z, Li Z-z, Zhu K-y, Dai F, Liu H-z, Zhang T-y, Sun C-z. Elevation of plasma tRNA fragments as a promising biomarker for liver fibrosis in nonalcoholic fatty liver disease. Scientific reports 2021; 11:5886
3. Tzur Y, Winek K, Madrer N, Dubnov S, Bennett ER, Greenberg DS, Hanin G, Gammal A, Tam J, Arkin IT, Paldor I, Soreq H. Lysine tRNA fragments and miR-194-5p co-regulate hepatic steatosis via β-Klotho and perilipin 2. Molecular metabolism 2024; 79:101856
4. Hardesty JE, Wahlang B, Falkner KC, Shi H, Jin J, Zhou Y, Wilkey DW, Merchant ML, Watson CT, Feng W, Morris AJ, Hennig B, Prough RA, Cave MC. Proteomic Analysis Reveals Novel Mechanisms by Which Polychlorinated Biphenyls Compromise the Liver Promoting Diet-Induced Steatohepatitis. Journal of proteome research 2019; 18:1582-1594
5. Petri BJ, Piell KM, Wahlang B, Head KZ, Andreeva K, Rouchka EC, Pan J, Rai SN, Cave MC, Klinge CM. Multiomics analysis of the impact of polychlorinated biphenyls on environmental liver disease in a mouse model. Environmental Toxicology and Pharmacology 2022; 94:103928
6. Andrews S. FastQC: A Quality Control Tool for High Throughput Sequence Data. 2014; http://bioinformatics.babraham.ac.uk/projects/fastqc/.
